# Cytokine production and phenotype of *Histomonas meleagridis*-specific T cells in the chicken

**DOI:** 10.1186/s13567-019-0726-z

**Published:** 2019-12-05

**Authors:** Julia Lagler, Taniya Mitra, Selma Schmidt, Alix Pierron, Eleni Vatzia, Maria Stadler, Sabine E. Hammer, Kerstin H. Mair, Beatrice Grafl, Patricia Wernsdorf, Fabienne Rauw, Bénédicte Lambrecht, Dieter Liebhart, Wilhelm Gerner

**Affiliations:** 1 Department of Pathobiology, Institute of Immunology, University of Veterinary Medicine Vienna, Vienna, Austria; 2 Department for Farm Animals and Veterinary Public Health, Clinic for Poultry and Fish Medicine, University of Veterinary Medicine Vienna, Vienna, Austria; 3 Avian Virology & Immunology Unit, Sciensano, Brussels, Belgium

## Abstract

The protozoan parasite *Histomonas meleagridis* is the causative agent of the re-emerging disease histomonosis of chickens and turkeys. Due to the parasite’s extracellular occurrence, a type-2 differentiation of *H. meleagridis*-specific T cells has been hypothesized. In contrast, a recent study suggested that IFN-γ mRNA^+^ cells are involved in protection against histomonosis. However, the phenotype and cytokine production profile of *H. meleagridis*-specific T cells still awaits elucidation. In this work, clonal cultures of a virulent monoxenic strain of *H. meleagridis* were used for infecting chickens to detect IFN-γ protein and IL-13 mRNA by intracellular cytokine staining and PrimeFlow™ RNA Assays, respectively, in CD4^+^ and CD8β^+^ T cells. Infection was confirmed by characteristic pathological changes in the cecum corresponding with *H. meleagridis* detection by immunohistochemistry and *H. meleagridis*-specific antibodies in serum. In splenocytes stimulated either with *H. meleagridis* antigen or PMA/ionomycin, IFN-γ-producing CD4^+^ T cells from infected chickens increased in comparison to cells from non-infected birds 2 weeks and 5 weeks post-infection. Additionally, an increase of IFN-γ-producing CD4^−^CD8β^−^ cells upon *H. meleagridis* antigen and PMA/ionomycin stimulation was detected. Contrariwise, frequencies of IL-13 mRNA-expressing cells were low even after PMA/ionomycin stimulation and mainly had a CD4^−^CD8β^−^ phenotype. No clear increase of IL-13^+^ cells related to *H.* *meleagridis* infection could be found. In summary, these data suggest that *H.* *meleagridis* infection induces a type-1 differentiation of CD4^+^ T cells but also of non-CD4^+^ cells. This phenotype could include γδ T cells, which will be addressed in future studies.

## Introduction

*Histomonas meleagridis* causes histomonosis (synonyms: enterohepatitis or blackhead disease) of chickens (*Gallus gallus*) and turkeys (*Meleagris gallopavo*) [1, 2]. The primarily affected organ is the cecum with generalized mucosal bleedings and severe fibrinous inflammation. Migration of the parasite through the portal vein leads to pathogenic changes in the liver characterized by multifocal areas of necrotic lesions. The clinical manifestation shows great variability among both species. While histomonosis in chickens usually leads to a decrease in weight gain and a drop in egg production, turkeys suffer more often with fatal outcome, as summarized previously [3]. Today, most countries impose a ban on previously applied prophylactic and therapeutic drugs due to consumer safety regulations. Because of these currently limited possibilities for medical intervention in combination with the high mortality in turkeys, histomonosis can cause severe suffering of infected birds. Hence, the disease is considered as a substantial economic threat for the poultry industry and requires the development of novel control strategies [4].

In order to protect turkeys from histomonosis, the transfer of antibodies was not effective [5]. Also, immunization by inactivated vaccines could not reduce mortality or lesions in turkeys [6, 7]. Instead, vaccination with a clonal in vitro attenuated *H. meleagridis* strain seems to be a promising strategy for protection of turkeys against histomonosis [7]. No reversion to virulence was observed so far for this in vitro attenuated *H. meleagridis* strain [8]. However, vaccine-induced immunity and protective traits against histomonosis are not fully understood yet. Upon *H. meleagridis* infection, a less profound influx of T cells in the liver of chickens compared to turkeys was observed [9]. A study by Mitra et al. [10] which analyzed different immune cell subsets in various organs, demonstrated distinct changes of B- and T-cell subsets after infection of chickens and turkeys, which were less pronounced in birds that had first undergone vaccination.

Due to the parasite’s extracellular occurrence, a type-2 immune response is conceivable. Powell et al. [9] studied a broad set of innate pro-inflammatory and adaptive cytokines on the mRNA level in the cecal tonsil and liver. In both organs from infected chickens, IFN-γ mRNA was up-regulated during the early stage of infection. In contrast, IL-13 mRNA expression was enhanced permanently. Another study investigated the immune response after co-infection of *H. meleagridis* and *Heterakis gallinarum* in chickens [11]. Detection of cytokines in the cecum by quantitative RT-PCR showed that infection without histomonads led to a type-2 dominated response characterized by IL-13 mRNA. Contrariwise, a co-infection resulted in a shift towards a type-1 dominated response with increased cecal IFN-γ mRNA expression. Recently the immune response against *H. meleagridis* was investigated by in situ hybridization (ISH) to detect cytokine transcript containing cells. Infected compared to vaccinated and challenged turkeys showed a delayed increase of IFN-γ mRNA^+^ cells in the cecum coinciding with severe tissue damage. Hence, an early rise in IFN-γ mRNA^+^ cells following vaccination could be protective by a rapid activation of effector memory T cells in turkeys [12].

None of these studies exclusively addressed *H. meleagridis*-specific T cells by T-cell markers. Therefore, we aimed to establish assays for identifying signature Th1 and Th2 cytokines in liver and spleen of chickens infected with clonal cultures of a virulent monoxenic strain of *H. meleagridis*. IFN-γ protein^+^ and IL-13 mRNA^+^ CD4^+^ and CD8β^+^ T cells were evaluated using *H. meleagridis* antigen as well as PMA/ionomycin stimulation by intracellular cytokine staining (ICS) and PrimeFlow™ RNA Assays (Thermo Fisher Scientific, Waltham, MA, USA), respectively. Our results indicate that *H. meleagridis* infection in chickens leads to IFN-γ but not IL-13 production in CD4^+^ T cells as well as non-CD4^+^ cells in liver and spleen, providing further evidence that this protozoan infection causes predominantly type-1 immune responses.

## Materials and methods

### Birds

Embryonated specific pathogen free (SPF) layer eggs (VALO, BioMedia, GmbH, Osterholz-Scharmbeck, Germany) were incubated and hatched at the Clinic for Poultry and Fish Medicine, University of Veterinary Medicine Vienna, Austria. After hatch, 12 chicks were placed in pens on wood shavings in rooms with filtered air under negative pressure. Feed and water were provided ad libitum.

### Preparation of cultures for infection

For infection of the birds, the previously established clonal culture *H. meleagridis*/Turkey/Austria/2922-C6/04 (23 passages) obtained by micromanipulation and co-cultivated with the bacterial strain *E. coli* DH5α was used [13, 14]. For control birds, an inoculum containing the bacterial strain *E. coli* DH5α alone with a defined concentration close to the infection inoculum was prepared. For that, an average *E. coli* DH5α concentration of 1 × 10^8^ CFU/mL (colony forming units) in three separate 72-h parasite cultures was determined. The cultivation medium contained Medium 199 with Earle’s salts, l-glutamine, 25 mM HEPES and l-amino acids (Gibco™, Thermo Fisher Scientific), 15% fetal calf serum (FCS) (Gibco™, Thermo Fisher Scientific) and 0.25% sterilized rice starch (Carl Roth, Karlsruhe, Germany). Counting of viable *H. meleagridis* cells was performed using Trypan Blue and a Neubauer hemocytometer (Sigma-Aldrich, St. Louis, MO, USA) to adjust the relevant number of the parasite for inoculation. For *E. coli* DH5α, CFUs were determined by counting *E. coli* serial dilutions on Coliform agar plates after an incubation at 37 °C for 24 h.

### Infection

At an age of 28 days, the chickens were separated into two groups (*n* = 6 per group, Additional file 1) in different rooms. All chickens were subcutaneously tagged for identification (Swiftach^®^, Avery Dennison, Glendale, CA, USA). Prior to infection, body weight, blood samples and cloacal swabs were taken. Subsequently, one group of birds was inoculated with 6 × 10^5^ cells of *H. meleagridis* co-cultured with 6 × 10^6^ CFU of the bacterial strain *E. coli* DH5α in 600 µL cultivation medium. The inoculum was equally split for application via the oral and cloacal route using a syringe with crop tube or a conventional 1 mL pipette (Eppendorf AG, Hamburg, Germany), respectively. Control birds were inoculated using the same administration routes with 1 × 10^8^ CFU of *E. coli* DH5α in 600 µL cultivation medium without the presence of *H. meleagridis*. After inoculation, birds were kept feed and water restricted for 5 h. Three birds of each group were sacrificed 2 weeks post-infection (pi) (14 days post-infection (dpi), 15 dpi, 16 dpi) and 5 weeks pi (37 dpi, 38 dpi, 39 dpi) (Additional file 1). The infection experiment was approved by the institutional ethics and animal welfare committee and the national authority according to §§ 26 ft. of Animal Experiments Act, Tierversuchsgesetz 2012-TVG 2012 (license number 68.205/0161-WFN/3b/2017).

### Clinical examination, necropsy and sampling

All birds were examined daily for clinical signs characteristic for histomonosis including depression, diarrhea and ruffled feathers. Once per week the body weight of the birds was measured and blood samples were collected, starting at the day of infection until the day of killing. Cloacal swabs were taken 3 times per week from the day of infection onwards. The cloacal swabs were subsequently transferred to 2 mL microtubes (Eppendorf AG) filled with 1.5 mL cultivation medium (composition of medium described above) for re-isolation of viable parasites and incubated at 40 °C. The re-isolations were evaluated daily using a microscope up to 5 days post sampling for the presence of *H.* *meleagridis*. Blood samples were stored overnight at 4 °C before centrifugation at 3300 × *g* for 12 min to obtain serum. The serum samples were stored at −20 °C before further application. For euthanasia, thiopental (medicamentum pharma GmbH, Allerheiligen im Mürztal, Austria) was applied intravenously and the birds were bled to death before necropsy and gross pathology were performed. Lesions in cecum and liver were determined according to a previously established lesion scoring (LS) system [15, 16]: LS 0 represents no lesion, whereas LS 1 to 4 indicates mild to severe pathological changes. Tissue samples of cecum and liver were preserved in formalin for detection of *H. meleagridis* using immunohistochemistry (IHC), as described below. The spleens and the remaining tissue of livers were transferred to beakers containing ice-cold phosphate buffered saline (PBS) + 2% FCS (both Gibco™, Thermo Fisher Scientific) prior to subsequent isolation of lymphocytes (see below).

### Detection of *H. meleagridis* by ELISA and immunohistochemistry

For detection of antibodies against *H. meleagridis,* an indirect sandwich ELISA was performed following a previously established protocol [17]. In brief, a rabbit anti-*Histomonas* serum at a dilution of 1:10 000 was used for coating the ELISA plate. After adding a blocking buffer (Thermo Fisher Scientific) to prevent unspecific binding, the histomonad antigen was added. Subsequently, the plate was incubated with the serum samples followed by incubations with the goat anti-chicken IgG-horseradish peroxidase (Southern Biotech, Birmingham, AL, USA) and the tetramethylbenzidine substrate solution (Calbiochem, Merck KGaA, Darmstadt, Germany). After stopping the reaction with sulphuric acid the positivity of the samples was determined on a defined cut-off value set at 0.54 which is based on optical densities (OD) measured at a wavelength of 450 nm according to the before mentioned publication.

IHC for the direct detection of *H.* *meleagridis* in tissues was applied as described by Singh et al. [18]. Briefly, after fixation, dehydration and embedding in paraffin, cuts of 4 µm in size were prepared using a microtome (Microm HM 360, Microm Laborgeräte GmbH, Walldorf, Germany) and transferred to glass slides (Superfrost plus, Menzel-Gläser, Braunschweig, Germany). After dewaxing and rehydration, samples were incubated overnight at 4 °C with a purified polyclonal anti-histomonad rabbit antibody. A biotinylated anti-rabbit IgG antibody (Vector Laboratories, Burlingame, CA, USA) was added after washing with PBS (Gibco™, Thermo Fisher Scientific). Following another washing step, the Vectastain ABC Kit and DAB Substrate Kit (Vector Laboratories) were used for visualizing *H. meleagridis*. The surrounding tissue was counterstained using Haematoxylin (Merck KGaA).

### Preparation of *H. meleagridis* and *E. coli* antigen stocks for in vitro re-stimulation

For in vitro re-stimulation of lymphocytes, a *H. meleagridis*/*E. coli* antigen stock as well as an *E. coli* control antigen stock were prepared as follows. The *H. meleagridis* with *E. coli* antigen was generated from the same clonal culture of *H. meleagridis*/Turkey/Austria/2922-C6/04 (23 passages) as used for infection. The *E. coli* antigen was prepared from the *E. coli* inoculum for the control birds (see above for cultivation conditions). The *H. meleagridis* with *E. coli* culture and the culture with *E. coli* alone were washed and resuspended with PBS (Gibco™, Thermo Fisher Scientific) at 200 × *g* for 5 min and at 1780 × *g* for 5 min, respectively, in order to remove the cultivation medium. The concentration of the *H. meleagridis* preparation was 9.25 × 10^6^ *H. meleagridis*/mL with 1 × 10^7^ *E. coli* CFU/mL and the *E.* *coli* alone preparation was 1.3 × 10^9^ CFU/mL. A freezing/thawing procedure at −80 °C was applied for three times. For removal of rice starch particles, a centrifugation step at 375 × *g* for 3 min was conducted. The supernatant was collected, aliquoted and frozen at −80 °C until used for stimulation.

### Isolation of lymphocytes

For isolation of lymphocytes, spleen and liver initially collected in PBS + 2% FCS were transferred into petri dishes. The splenic capsule was removed and tissue teased apart by using two sterile blunt-end forceps. Liver tissue was dissected by squeezing the tissue with the end of a plunger from a 20 mL syringe. Cell suspensions from both organs were filtered through 40 µm cell strainers (BD Falcon™, BD Biosciences, San Jose, CA, USA). Spleen and liver cell suspensions were centrifuged at 350 × *g* for 10 min at room temperature and the supernatant was discarded. The cell pellet was resuspended with cold PBS + 2% FCS and layered on a double volume of Histopaque^®^-1077 (Sigma-Aldrich). After centrifugation at 850 × *g* for 20 min at room temperature, the interphase was collected. Following washing with PBS and centrifugation at 650 ×* g* for 10 min at 4 °C, cells were resuspended in PBS and stored on ice until further processing. Cell viability and counting was performed using Trypan Blue and a Neubauer hemocytometer (Sigma-Aldrich).

### Establishment of intracellular cytokine staining for chicken IFN-γ

A panel of six monoclonal antibodies (mAbs) against chicken IFN-γ were investigated for their suitability in intracellular cytokine staining. Four mouse IgG1 (2B7, 11G5, 7E3, 12F12), one mouse IgG2a (12F7) and one mouse IgG2b (12D4) mAb clone were tested. Chicken splenocytes were seeded at 5 × 10^5^ cells per well into 96-well round-bottom microtiter plates (Greiner Bio-One, Kremsmünster, Austria) in 200 µL RPMI 1640 (PAN Biotech GmbH, Aidenbach, Germany) supplemented with stable glutamine, 10% heat inactivated FCS (Sigma-Aldrich), 100 IU/mL penicillin and 0.1 mg/mL streptomycin (PAN Biotech GmbH). Splenocytes were cultivated overnight in a humidified incubator at 41 °C and 5% CO_2_. The following day the microcultures were treated with PMA (50 ng/mL, Sigma-Aldrich) and ionomycin (500 ng/mL, Sigma-Aldrich) in the presence of Brefeldin A (1 µg/mL; BD GolgiPlug™, BD Biosciences) and incubated for additional 4 h. Following harvest, cells were washed twice in PBS (470 × *g* for 4 min at 4 °C; also used for all subsequent washing steps) and surface stained in 96-well plates with mAbs for CD4 (clones: 2–35, mouse IgG2b, Bio-Rad Laboratories, Hercules, CA, USA or CT-4, mouse IgG1, Southern Biotech; both FITC-conjugated) and the Fixable Viability Dye eFluor^®^ 780 (Thermo Fisher Scientific) for 20 min at 4 °C. Afterwards, cells were washed with PBS + 2% FCS. For fixation and permeabilization, the BD Cytofix/Cytoperm (BD Biosciences) kit was applied according to the manufacturer’s instructions. After this, the cells were incubated with the mAbs specific for IFN-γ mentioned above. Each antibody was tested in log2 serial dilutions, starting from 200 to 6.25 ng per sample. Following two washing steps with BD Perm/Wash™ Buffer (BD Biosciences), cells were incubated either with goat anti-mouse IgG1-RPE, IgG2a-RPE or IgG2b-RPE secondary antibodies (Southern Biotech) depending on the isotype of the IFN-γ specific mAb. Both incubation steps were performed for 30 min at 4 °C. Finally, the stained cells were washed twice, resuspended in 200 µL BD Perm/Wash™ Buffer (BD Biosciences) and transferred into 5 mL tubes for flow cytometry (FCM) analysis.

### Ectopic expression of chicken IL-13 in HEK293T cells

For scrutinizing the suitability of the IL-13 PrimeFlow™ RNA Assay (Thermo Fisher Scientific) for the detection of chicken IL-13 mRNA, immortalized human epithelial 293 kidney cells (HEK293T; originally provided by K. Vanura, Medical University Vienna, Austria) were transfected with chicken IL-13 DNA inserted in a pFLAG-CMV2 expression vector (Sigma-Aldrich) by directional cloning. As control, HEK293T cells were transfected with irrelevant porcine IgE. A detailed protocol on this is given in Additional file 2.

### In vitro stimulation of cells and intracellular cytokine staining

Lymphocytes from spleen and liver were cultivated under the conditions given above. For intracellular IFN-γ staining, cells were either stimulated overnight with 100 µL of *H. meleagridis* (5 × 10^4^/mL) with *E. coli* (5.4 × 10^4^ CFU/mL), *H. meleagridis* (5 × 10^3^/mL) with *E. coli* (5.4 × 10^3^ CFU/mL), *E.* *coli* (9.4 × 10^6^ CFU/mL) alone, *E. coli* (9.4 × 10^5^ CFU/mL) alone, or kept in culture medium as a negative control. An approximately 150 times higher bacterial concentration for the *E. coli* alone antigen than used for the *H.* *meleagridis* with *E. coli* antigen was chosen to reach similar protein levels within both preparations. Protein concentrations were determined by a Bradford Assay according to the manufacturer’s instructions (Bio-Rad). The *H. meleagridis* with *E. coli* preparation had a protein concentration of 70 µg/mL. For re-stimulation, the preparation was diluted to a protein concentration of 10 µg/mL, which was equal to 5 × 10^4^ histomonads cells/mL and 5.4 × 10^4^ *E. coli* CFU/mL. The same calculation was performed for the *E. coli* alone preparation resulting in a concentration of 9.4 × 10^6^ CFU/mL. As positive control, one set of wells was treated with 20 µL of PMA (50 ng/mL, Sigma-Aldrich) and ionomycin (500 ng/mL, Sigma-Aldrich) for 4 h. Per stimulation group, six wells with 5 × 10^5^ cells/well were seeded. The Golgi-inhibitor Brefeldin A was added to all stimulation groups during the final 4 h of stimulation at a concentration of 1 µg/mL (BD GolgiPlug™, BD Biosciences). For the IL-13 PrimeFlow™ RNA Assay (Thermo Fisher Scientific), the same stimulation variants were prepared as described above for IFN-γ with the exception of applying two different time spans for antigen-specific re-stimulation. Cells of three birds from each inoculation group were stimulated overnight (for both infected and control group: one bird from the first necropsy 2 weeks pi and two birds from the second necropsy 5 weeks pi) whereas the cells of the remaining three birds were stimulated for 4 h (opposite distribution of birds compared to overnight stimulation). Data obtained from both time spans are grouped together in the results section, since no obvious differences related to the time of stimulation were observed.

Afterwards, cells were pooled and washed as described above. For surface staining, cells were incubated with mouse anti-chicken CD4-FITC (clone: 2–35, isotype: IgG2b, Bio-Rad Laboratories) and biotinylated CD8β (clone: EP42, isotype: IgG2a, Southern Biotech) mAbs for 20 min at 4 °C. In a second staining step, Streptavidin eFluor™ 450 (Thermo Fisher Scientific) together with Fixable Viability Dye eFluor^®^ 780, (Thermo Fisher Scientific) was applied (20 min, 4 °C). For intracellular IFN-γ staining, the BD Cytofix/Cytoperm (BD Biosciences) kit was used for fixating and permeabilizing cells. Chicken IFN-γ-specific mAb 11G5, isotype mouse IgG1, was added and further labelled by goat anti-mouse IgG1-RPE (Southern Biotech). Washing steps were performed as described above. For IL-13 mRNA staining, the PrimeFlow™ RNA Assay kit (Thermo Fisher Scientific) was employed according to the manufacturer’s instructions. The target probe specific for chicken IL-13 mRNA was designed by the company based on the sequence accession number NM_001007085 (Assay ID: VF1-4170930-PF, Thermo Fisher Scientific). An Alexa Fluor™ 647-conjugated label probe for detection of the target probe was selected. Cell surface staining as well as staining with Fixable Viability Dye eFluor^®^ 780 (Thermo Fisher Scientific) was performed as described above for IFN-γ labelling. Thereafter, cells were fixated for 30 min at 4 °C, washed twice with permeabilization buffer and fixated for a second time for 60 min at room temperature. In between, a centrifugation step with 1000 × *g* for 4 min at 4 °C was applied. Afterwards, cells were incubated with the chicken IL-13 mRNA specific target probe for 2 h at 41 °C. Followed by two washes at room temperature and adding 100 µL PrimeFlow™ RNA Assay wash buffer (Thermo Fisher Scientific), cells were stored overnight at 4 °C in the dark. After two amplification steps for 1.5 h at 41 °C, cells were incubated with the fluorescence labelled probe for 1 h at 41 °C. After three washing steps, cells were resuspended in 200 µL PrimeFlow™ RNA Assay storage buffer (Thermo Fisher Scientific) using 5 mL tubes for subsequent FCM-analysis.

### Cell analysis by flow cytometry

For measurement of IFN-γ protein stained cells and for IL-13 mRNA stained cells, a FACSAria and a FACSCanto II (both BD Biosciences) were used, respectively. Both flow cytometers were equipped with three lasers (405, 488, 633 nm). Between 2 × 10^5^ and 6 × 10^5^ lymphocytes (identified by light scatter properties) were acquired per sample. Flow cytometry data was acquired by FACSDiva software version 6.1.3 (BD Biosciences) and analyzed by FlowJo™ software (Version 10.5.0, Tree Star, Ashland, OR, USA).

### Processing of results and statistical analysis

For a calculation of the frequency of *H. meleagridis*-specific T cells, percentages of cytokine-producing (IFN-γ or IL-13) lymphocyte subsets from *E. coli*-only stimulated samples were subtracted from percentages of *H. meleagridis*/*E. coli* co-stimulated samples. To determine significant differences in cytokine-producing cell subsets (IFN-γ or IL-13) between the two necropsies (14–16 dpi and 37–39 dpi), a Wilcoxon test was applied. Differences between stimulated and non-stimulated cytokine-producing cell subsets from the same bird were subjected to the Wilcoxon test as well. Mann–Whitney tests were applied to compare cytokine-producing cell subsets isolated from control birds with cytokine-producing cell subsets isolated from infected birds. *p*-values < 0.05 are indicated by * and *p*-values < 0.01 are indicated by **. Statistical analyses were performed by the GraphPad Prism software 7.04 (GraphPad Software Inc., San Diego, CA, USA).

## Results

### Establishment of test systems for the detection of IFN-γ protein by ICS and IL-13 mRNA by PrimeFlow RNA™

To expand the chicken toolbox on antibodies suitable for the detection of IFN-γ by ICS, six monoclonal antibodies, initially successfully applied in ELISA [19], were investigated. Following stimulation of splenocytes with PMA/ionomycin, four out of six monoclonal antibodies (clones: 12F7, 2B7, 11G5, 7E3) identified similar frequencies (2.39 to 2.88%) of IFN-γ-producing cells (Additional file 3, the applied gating is illustrated in Additional file 4A). Predominantly, CD4^+^ cells produced IFN-γ. Monoclonal antibody clone 12D4 detected 0.84% IFN-γ^+^ cells within live lymphocytes while clone 12F12 seemed to be not suitable for ICS since hardly any IFN-γ^+^CD4^+^ splenocytes were found. For all subsequent experiments clone 11G5 with a mouse IgG1 isotype was selected.

As outlined in “Materials and methods”, PrimeFlow™ RNA Assays were applied in order to detect chicken IL-13 mRNA on the single cell level by flow cytometry. The test was performed according to the manufacturer’s instructions but we scrutinized its specificity by the use of HEK293T cells, which were transfected for expression of chicken IL-13 (Additional files 5A and B). The gating strategy applied in these experiments is illustrated in Additional file 5A. The IL-13 specific target probe could detect IL-13 mRNA-expressing cells (33.6%), while staining without the target probe but only the label probe revealed no such population (0.02%; Additional file 5B, upper row). The labelling of HEK293T cells transfected with the same vector but containing an insert that codes for porcine IgE also led to no IL-13^+^ cells neither in presence (0.01%) nor in absence (0%) of the target probe (Additional file 5B, lower row). Hence, both methods appeared to be reliable and were subsequently applied in a controlled infection experiment of chickens with *H. meleagridis* (see Additional file 1 for outline of the experiment).

### Clinical signs, pathological score, *H. meleagridis*-specific IHC and circulating antibodies

No clinical signs were detected in any bird from the infected and control group. Pathological lesion scores (LS) of cecum and liver determined during post-mortem necropsy are summarized in Table 1. In the ceca, a maximum LS of 4 was reached for one infected bird at 2 weeks pi and overall the scores decreased for birds sacrificed 5 weeks pi to 2. Ceca collected 2 weeks pi and 5 weeks pi reached a median LS of 3 and 2, respectively. Livers of birds in the infected group were scored and the median LS was zero at both time spans, i.e. 2 weeks pi and 5 weeks pi. The non-infected control birds did not show any lesion in cecum and liver. In two out of six infected birds *H. meleagridis* could be re-isolated from cloacal swab samples (bird 11: 32 and 37 dpi; bird 12: 25, 28 and 30 dpi).

**Table 1:** **Pathological changes and** ***H. meleagridis*** **detection in the cecum and liver**

	Days post-infection	Animal number	Cecum		Liver	
			LS^a^	IHC^b^	LS	IHC
Control	14	1	0	−	0	−
	15	2	0	−	0	−
	16	3	0	−	0	−
	37	4	0	−	0	−
	38	5	0	−	0	−
	39	6	0	−	0	−
Infected	14	7	3	+	0	−
	15	8	4	−	0	−
	16	9	2	+	0	−
	37	10	2	+	0	−
	38	11	2	+	0	−
	39	12	2	+	2	−

^a^Lesion scoring (LS) system from 0 to 4 was applied; Cecum: 0 = no pathological changes; 1 = sporadic inflammation and/or mild thickening of the wall of one cecum; 2 = sporadic inflammation and/or mild thickening of the wall of both ceca; 3 = inflammation of both ceca and thickening of the intestinal wall with liquid fibrin or sporadic fibrinous coagula in the lumen. If only one cecum was affected, then lesion score 2 was applied; 4 = severe inflammation and necrosis in both ceca with compact fibrinous masses in the lumen of the ceca. If only one cecum was affected, then lesion score 3 was applied. Liver: 0 = no pathological changes; 1 = a few single punctiform necrosis up to 1 mm; 2 = single punctiform necrosis disseminated throughout the organ up to 1 mm or a few single punctiform necrosis more than 1 mm; 3 = single punctiform necrosis, disseminated throughout the organ more than 1 mm or some large area necrosis; 4 = confluent necrosis throughout the organ.

^b^Detection of the parasite in cecum and liver was performed by immunohistochemistry.

Detection of *H. meleagridis* by IHC in ceca and livers is also depicted in Table 1. All infected birds were found positive in the cecum except one bird which showed the highest detected cecal LS. Livers of infected birds were negative, including one bird that reached a LS of 2. The control birds were confirmed to be non-infected by IHC.

Results from the testing for circulating antibodies against *H. meleagridis* in sera from birds are shown in Table 2. All non-infected chickens were tested negative at all sampling days. Birds of the infected group killed 2 weeks pi also stayed below the threshold for positivity at every time point of sampling. In contrast, two of the remaining infected birds showed positive antibody titers already 2 weeks pi and 5 weeks pi all three infected birds showed antibody titers above the cut-off.

**Table 2:** ***H. meleagridis*-specific antibodies in serum**

	Bird no.	Week post-infection					
		0	1	2	3	4	5
Control	1	−	−	−			
	2	−	−	−			
	3	−	−	−			
	4	−	−	−	−	−	−
	5	−	−	−	−	−	−
	6	−	−	−	−	−	−
Infected	7	−	−	−			
	8	−	−	−			
	9	−	−	−			
	10	−	−	+	−	−	+
	11	−	−	−	−	−	+
	12	−	−	+	−	+	+

− Indicates O.D. values below threshold of positivity.

+ Indicates O.D. values above threshold of positivity.

### T-cell response following *H. meleagridis* infection

**Figure 1:**
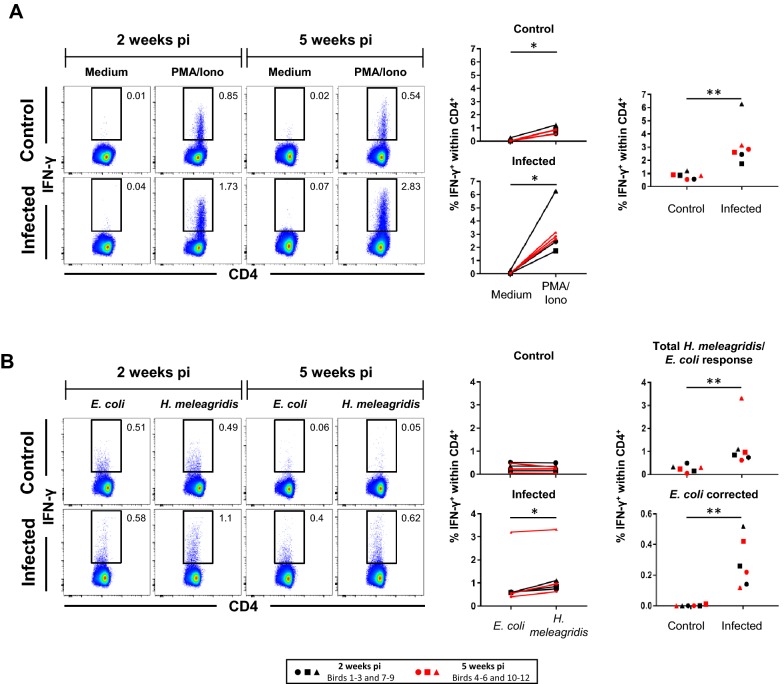
**Frequencies of splenic IFN-γ-producing CD4**^**+**^ **cells following stimulation with PMA/ionomycin or** ***H. meleagridis***. **A**, **B** Representative pseudocolor plots show IFN-γ versus CD4 expression in CD4^+^ pre-gated (not depicted) splenocytes isolated from birds 2 weeks pi and 5 weeks pi. Approximately **A** 200 000 and **B** 150 000 CD4^+^ cells are shown in each plot and numbers indicate frequencies of IFN-γ^+^CD4^+^ cells within total CD4^+^ cells. Graphs on the right display frequencies of IFN-γ-producing CD4^+^ cells from all birds. Each symbol represents one bird, black and red colored symbols represent birds sacrificed 2 weeks pi and 5 weeks pi, respectively. **A** Scatter plots show percent of IFN-γ^+^ cells within the CD4^+^ subset after PMA/ionomycin stimulation compared to medium in control and infected birds (left panel). Right panel: comparison of IFN-γ-producing CD4^+^ cell frequencies after stimulation with PMA/ionomycin between infected and control birds. **B** Scatter plots as in **A** but after *H. meleagridis*/*E. coli* stimulation and *E. coli*-only stimulation. Right panel shows in addition percent of IFN-γ^+^ cells after *E. coli* correction for infected and control birds. Asterisks indicate different *p*-values: *p ≤ 0.05 and **p ≤ 0.01.

**Figure 2:**
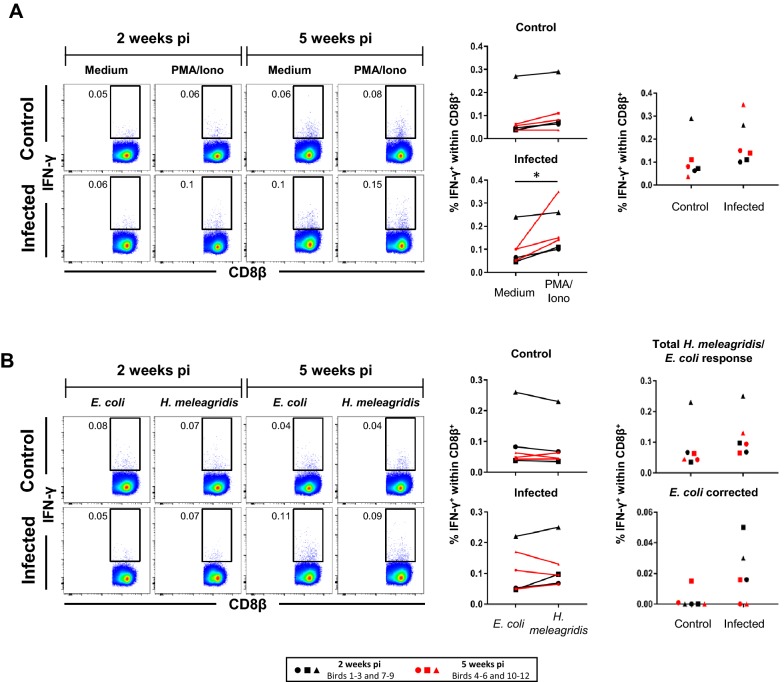
**Frequencies of splenic IFN-γ-producing CD8β**^**+**^ **cells following stimulation with PMA/ionomycin or** ***H. meleagridis***. **A**, **B** Representative pseudocolor plots show IFN-γ versus CD8β expression in CD8β^+^ pre-gated (not depicted) splenocytes isolated from birds 2 weeks pi and 5 weeks pi. Approximately **A** 270 000 and **B** 300 000 CD8β^+^ cells are shown in each plot and numbers indicate frequencies of IFN-γ^+^CD8β^+^ cells within total CD8β^+^ cells. Graphs on the right display frequencies of IFN-γ-producing CD8β^+^ cells from all birds. Each symbol represents one bird, black and red colored symbols represent birds sacrificed 2 weeks pi and 5 weeks pi, respectively. **A** Scatter plots show percent of IFN-γ^+^ cells within the CD8β^+^ subset after PMA/ionomycin stimulation compared to medium in control and infected birds (left panel). Right panel: comparison of IFN-γ-producing CD8β^+^ cell frequencies after stimulation with PMA/ionomycin between infected and control birds. **B** Scatter plots as in **A** but after *H. meleagridis*/*E. coli* stimulation and *E. coli*-only stimulation. Right panel shows in addition percent of IFN-γ^+^ cells after *E. coli* correction for infected and control birds. Asterisk indicates *p*-value: *p ≤ 0.05.

**Figure 3:**
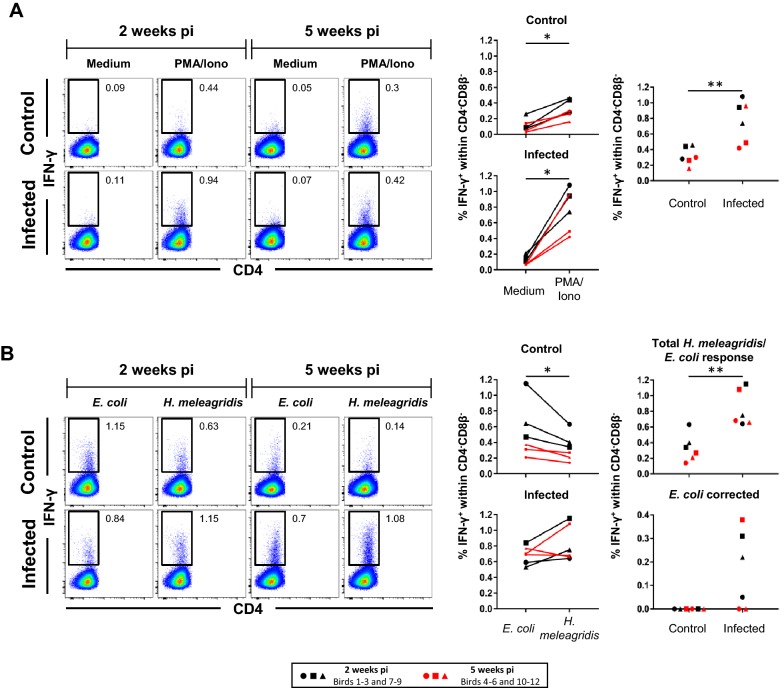
**Frequencies of splenic IFN-γ-producing CD4**^**−**^**CD8β**^**−**^ **cells following stimulation with PMA/ionomycin or** ***H. meleagridis***. **A**, **B** Representative pseudocolor plots show IFN-γ versus CD4 expression in CD4^−^CD8β^−^ pre-gated (not depicted) splenocytes isolated from birds 2 weeks pi and 5 weeks pi. Approximately **A** 200 000 and **B** 180 000 CD4^−^CD8β^−^ cells are shown in each plot and numbers indicate frequencies of IFN-γ^+^CD4^−^CD8β^−^ cells within total CD4^−^CD8β^−^ cells. Graphs on the right display frequencies of IFN-γ-producing CD4^−^CD8β^−^ cells from all birds. Each symbol represents one bird, black and red colored symbols represent birds sacrificed 2 weeks pi and 5 weeks pi, respectively. **A** Scatter plots show percent of IFN-γ^+^ cells within the CD4^−^CD8β^−^ subset after PMA/ionomycin stimulation compared to medium in control and infected birds (left panel). Right panel: comparison of IFN-γ-producing CD4^−^CD8β^−^ cell frequencies after stimulation with PMA/ionomycin between infected and control birds. **B** Scatter plots as in **A** but after *H. meleagridis*/*E. coli* stimulation and *E. coli*-only stimulation. Right panel shows in addition percent of IFN-γ^+^ cells after *E. coli* correction for infected and control birds. Asterisks indicate different *p*-values: *p ≤ 0.05 and **p ≤ 0.01.

**Figure 4:**
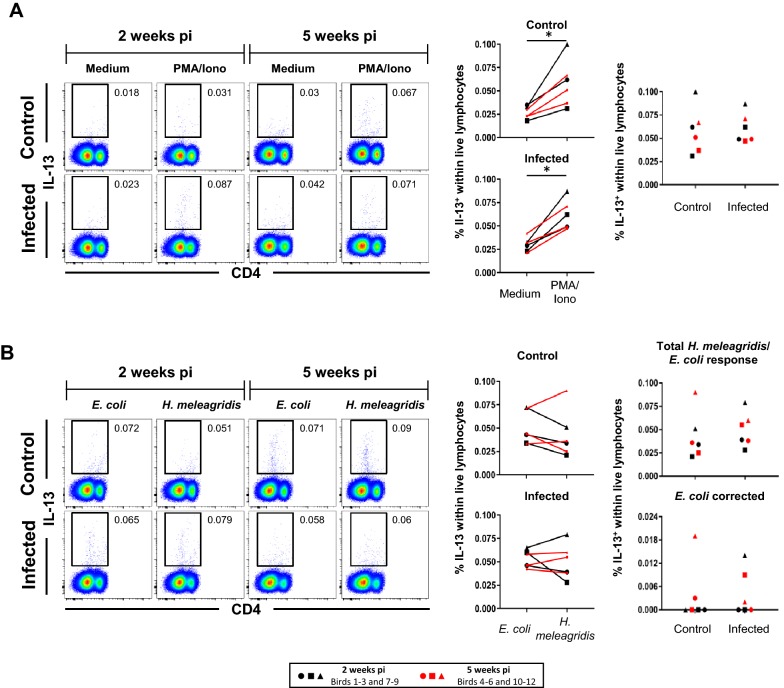
**Frequencies of splenic IL-13 mRNA**^**+**^ **cells following stimulation with PMA/ionomycin or** ***H. meleagridis***. **A**, **B** Representative pseudocolor plots show IL-13 mRNA versus CD4 expression in pre-gated (not depicted) total live splenocytes isolated from birds 2 weeks pi and 5 weeks pi. Approximately 320 000 lymphocytes are shown in each plot and numbers indicate frequencies of IL-13 mRNA^+^ cells within total live splenocytes. Graphs on the right display frequencies of IL-13 mRNA^+^ live lymphocytes from all birds. Each symbol represents one bird, black and red colored symbols represent birds sacrificed 2 weeks pi and 5 weeks pi, respectively. **A** Scatter plots show percent of IL-13 mRNA^+^ cells within live lymphocytes after PMA/ionomycin stimulation compared to medium in control and infected birds (left panel). Right panel: comparison of IL-13 mRNA^+^ lymphocyte frequencies after stimulation with PMA/ionomycin between infected and control birds. **B** Scatter plots as in **A** but after *H. meleagridis*/*E. coli* stimulation and *E. coli*-only stimulation. Right panel shows in addition percent of IL-13 mRNA^+^ cells after *E. coli* correction for infected and control birds. Asterisks indicate *p*-value: *p ≤ 0.05.

To investigate the T-cell response following *H. meleagridis* infection, two time spans of 14–16 dpi and 37–39 dpi were initially chosen to compare an early T-cell response with a later time point, where potentially an immune memory phase may already have been reached. However, an initial analysis of the obtained frequencies of IFN-γ protein or IL-13 mRNA-producing lymphocyte subsets suggested no major differences between these two time spans. To scrutinize this, Wilcoxon tests were applied and no significant differences were found between the two time spans. Hence, in all results shown in Figures 1, 2, 3, 4, phenotypes of cytokine-producing cells from these two necropsies were grouped together and subjected to a Mann–Whitney test for significance testing. In addition, in Figures 1, 2, 3, 4 original flow cytometry data of one representative bird per group is shown in pseudocolor plots on the left while percentages of cytokine-producing cells for all birds are illustrated on the right, including a comparison of the stimulation variants between infected and control animals. Underlying values of cytokine-producing lymphocytes in these analyses are listed in Additional file 6, together with the calculated *E. coli* corrected values.

### IFN-γ production in CD4/CD8β defined lymphocyte subsets in the spleens of *H. meleagridis* infected chickens

ICS analyses were performed for IFN-γ and combined with cell surface staining for CD4 and CD8β, allowing the identification of IFN-γ production in CD4^+^, CD8β^+^ and CD4^−^CD8β^−^ cells (gating strategy is illustrated in Additional file 4A). PMA/ionomycin stimulation led to a significant increase in IFN-γ-producing cells within total CD4^+^ splenocytes regardless whether cells derived from control (*p* < 0.05) or *H. meleagridis* infected chickens (*p* < 0.05; Figure 1A, scatter plots on the left). A significantly elevated level of IFN-γ^+^CD4^+^ cells after PMA/ionomycin stimulation was found in infected birds in comparison to control birds (*p* < 0.01; Figure 1A, scatter plot on the right). *H. meleagridis*/*E. coli* stimulation in comparison to *E. coli*-only control stimulation revealed significantly higher levels (*p* < 0.05) of IFN-γ^+^ cells within CD4^+^ splenocytes in infected birds (Figure 1B, scatter plot on the left, bottom). No such difference was detectable in control birds, which showed low frequencies of IFN-γ^+^ cells within the CD4^+^ subset in *H. meleagridis*/*E. coli* stimulated cultures and *E. coli*-only stimulated control cultures (Figure 1B, scatter plot on the left, top). Percentages of *H. meleagridis*/*E.* *coli* stimulated IFN-γ-producing cells were significantly higher in infected than in control birds (*p* < 0.01), also after correcting the data for IFN-γ-producing splenocytes induced by *E. coli*-only stimulation (*p* < 0.01; Figure 1B, scatter plots on the right). Ten-fold lower concentrations of *H. meleagridis* antigen (5 × 10^3^/mL) were also tested for the induction of IFN-γ-producing CD4^+^ splenocytes. Results are shown in comparison to data obtained with 5 × 10^4^ *H. meleagridis*/mL (Additional file 7). Although still elevated levels of IFN-γ^+^CD4^+^ cells in infected versus control birds were found (Additional file 7, scatter plots in right column of right panel), after correction of *E. coli* these differences did not reach significance.

For CD8β^+^ splenocytes (Figure 2) in control birds, no significant rise in IFN-γ-producing cells was detected upon PMA/ionomycin stimulation in comparison to non-stimulated cells. For infected birds, a significant increase for this condition was found (*p* < 0.05; Figure 2A, scatter plots on the left). However, although PMA/ionomycin-induced IFN-γ^+^CD8β^+^ splenocytes within total CD8β^+^ splenocytes from the infected group were in tendency higher than those ones in the control group, these differences did not reach significance (Figure 2A, scatter plot on the right). Similarly, *H. meleagridis* re-stimulation led to no significant difference between infected and control birds for IFN-γ^+^CD8β^+^ splenocytes. This applied also when the data was corrected for *E. coli*-only induced IFN-γ-producing CD8β^+^ splenocytes (Figure 2B, scatter plots on the right).

IFN-γ-producing cell frequencies within the remaining CD4^−^CD8β^−^ subset of splenocytes were also investigated (Figure 3). PMA/ionomycin stimulation versus medium led to a significant increase in IFN-γ^+^CD4^−^CD8β^−^ splenocytes in control and infected birds (both *p* < 0.05; Figure 3A, scatter plots on the left). Infected compared to control birds disclosed a significantly higher level of IFN-γ^+^CD4^−^CD8β^−^ cells in infected birds after PMA/ionomycin stimulation (*p* < 0.01; Figure 3A, scatter plot on the right). For most of the infected birds, higher levels of IFN-γ^+^ cells within CD4^−^CD8β^−^ splenocytes were identified upon *H. meleagridis*/*E.* *coli* stimulation compared to *E. coli*-only stimulated controls, but this did not reach significance (Figure 3B, scatter plots on the left). However, *H. meleagridis*/*E. coli* re-stimulation induced a significant difference between control and infected birds (*p* < 0.01) but significance was lost after a correction of data for *E. coli*-only induced IFN-γ production (Figure 3B, scatter plots on the right). Overall, these results indicate the generation of *H. meleagridis*-specific IFN-γ-producing splenocytes with a CD4^+^ and a CD4^−^CD8β^−^ phenotype in *H. meleagridis*-infected chickens. Interestingly, for these two phenotypes PMA/ionomycin stimulation also caused higher frequencies of IFN-γ-producing cells in *H. meleagridis* infected chickens than in control chickens.

### IFN-γ production in CD4/CD8β defined lymphocyte subsets in livers of *H. meleagridis* infected chickens

In parallel to the analyses with splenocytes, IFN-γ^+^ cell frequencies in livers were determined for CD4^+^, CD8β^+^ and CD4^−^CD8β^−^ T-cell subsets. In contrast to data obtained with splenocytes, neither in CD4^+^ nor in CD4^−^CD8β^−^ lymphocytes derived from the liver significant differences in the frequencies of IFN-γ-producing lymphocytes between *H*. *meleagridis* infected chickens and control chickens were found (Additional file 8, scatter plots in the lower part). This applied to both, PMA/ionomycin stimulation (Additional file 8, left columns of scatter plots) and *H. meleagridis* re-stimulation (Additional file 8, right columns of scatter plots). For CD8β^+^ cells in the liver, hardly any IFN-γ-producing cells were identified (IFN-γ^+^ cells ranged from 0 to 0.1%), regardless of the type of in vitro stimulation, treatment of the birds and time point of isolation post-infection (data not shown).

### IL-13 mRNA production of lymphocytes in the spleen of *H. meleagridis* infected chickens

Frequencies of IL-13 mRNA-producing lymphocytes were investigated by PrimeFlow™ RNA Assay (Thermo Fisher Scientific) in combination with cell surface staining for CD4 and CD8β. A gating strategy as depicted in Additional file 4B was applied for identifying IL-13 mRNA^+^ cell frequencies within live splenocytes. No further sub-gating for CD4 or CD8β expression was applied since IL-13 mRNA^+^ cells appeared to have a CD4^dim/−^CD8β^−^ phenotype (Additional file 4B and Figures 4A and B, pseudocolor plots on the left).

Stimulation with PMA/ionomycin compared to medium significantly increased the frequency of IL-13 mRNA^+^ splenocytes in infected (*p* < 0.05) and control birds (*p* < 0.05; Figure 4A, scatter plots on the left). Comparing values of PMA/ionomycin stimulated IL-13 mRNA^+^ cells revealed no obvious difference between infected and control birds (Figure 4A, scatter plot on the right). *E. coli*-only stimulated control cultures induced slightly higher levels of IL-13 mRNA^+^ cells than *H. meleagridis*/*E. coli* stimulated cultures in control birds, but this did not reach significance (Figure 4B, scatter plot on the left, top). Also in infected birds, no major difference in IL-13 mRNA^+^ cells was detected between *H. meleagridis*/*E. coli* and *E. coli*-only stimulation (Figure 4B, scatter plot on the left, bottom). Frequencies of IL-13 mRNA^+^ splenocytes in infected birds did not significantly differ from frequencies in control animals upon *H. meleagridis*/*E. coli* stimulation and after correcting data from *E. coli* background (Figure 4B, scatter plots on the right). Overall, frequencies of IL-13 mRNA-producing cells detected by flow cytometry were very low and showed no clear phenotype for CD4 or CD8β expression.

## Discussion

In avian species, the immunological toolbox for studying cytokine production of different T-cell subsets, especially antigen-specific T cells, is still limited. Flow cytometry-based intracellular cytokine staining is a powerful tool for investigating the frequency and phenotype of cytokine-producing immune cells [20]. The detection of IFN-γ by ICS following in vitro stimulation has only been applied in a limited number of studies using chicken IFN-γ specific mAb clones mAb80 [21], EH9 [22] or the coating and detection antibody of the IFN-γ CytoSet™ ELISA (Thermo Fisher Scientific) [23]. In the present study, six IFN-γ monoclonal antibodies were screened for their capability of detecting chicken IFN-γ by ICS following PMA/ionomycin stimulation (Additional file 3). Four of them detected similarly high frequencies of IFN-γ-producing splenocytes and one of them has a mouse IgG2a phenotype, making it an attractive candidate in multicolor staining panels where isotype-specific secondary antibodies are in use. Hence, the aforementioned studies and our data indicate that there is a panel of IFN-γ-specific antibodies, which are all suitable for ICS in chicken lymphocytes.

In our study, detection of IFN-γ by ICS was tested alongside to detection of IL-13 mRNA by PrimeFlow™ RNA Assay (Thermo Fisher Scientific). Both assays were applied to investigate cytokine production in spleen and liver derived lymphocytes from *H.* *meleagridis* infected chickens. *H. meleagridis*-specific antibodies, which were investigated in parallel to the T-cell response, showed some degree of variability among infected chickens (Table 2). However, this variability is in accordance with previously published data from Windisch and Hess [15], where a very similar infection model as in our study was applied. Rather, this variability might be seen as an indication that analyses on the T-cell response will contribute to a better understanding of the immune response in chickens against *H.* *meleagridis*. Indeed, against the hypothesis of a dominating type-2 immune response following an extracellular parasitic infection, which might also support antibody production, our data suggest a type-1 response mainly driven by IFN-γ-producing CD4^+^ T cells (see also summary of significantly increased cytokine-producing lymphocytes subsets in Additional file 9). Significant rises of putative *H. meleagridis*-specific T cells in the spleen with an IFN-γ^+^CD4^+^ phenotype could be found upon antigen re-stimulation in infected compared to non-infected control birds. This did not apply to CD8β^+^ splenocytes, in which after both, PMA/ionomycin and *H. meleagridis* stimulation, only low frequencies of IFN-γ-producing cells were found and frequencies in infected chickens were only slightly above control chickens, not reaching significance. This suggests that at least for chickens, conventional CD8 T cells are not involved in the sytemic immune response against *H. meleagridis*, which might be explained by the extracellular occurrence of the parasite.

Next to CD4^+^ cells, significant enhancements of IFN-γ-producing splenocytes within the CD4^−^CD8β^−^ subset upon both stimulation approaches in infected birds could be identified. This finding suggests an involvement of putative γδ T cells or NK cells. Chicken γδ T cells are highly abundant in blood as well as in lymphatic organs and can be divided according to their CD8 expression into CD8^−^, CD8α^hi^β^+^ and CD8αα^hi+^ subpopulations [24, 25]. Splenic CD8αα^+^ γδ T cells seem to be the most potent IFN-γ producers among all γδ T-cell subsets [26]. Published data on the transcription levels of IFN-γ in *Salmonella* Typhimurium infected chickens identified γδ T cells as a possible source of IFN-γ as well [27]. In the last mentioned study, significant increases of IFN-γ mRNA in γδ T cells with CD8αα^+^ and CD8α^+^β^+^ phenotypes from blood and spleen were observed. Besides γδ T cells, NK cells are known to be major IFN-γ producers in various mammalian species [28, 29]. However, in chickens, NK cells seem to account only for a minor lymphocyte subpopulation in blood and spleen with frequencies below 3%; albeit some of them have the capacity for IFN-γ production [30, 31]. Clearly, a more precise phenotyping of the identified *H. meleagridis*-specific IFN-γ-producing CD4^−^CD8β^−^ lymphocytes is something that we will aim to address in future studies.

On the contrary, frequencies of IL-13-producing lymphocytes derived from spleen and liver were extremely low and did not rise following stimulation with *H.* *meleagridis* antigen. So far, only studies on the gene expression of various cytokines from *H.* *meleagridis* infected chickens and turkeys were published. Powell et al. [9] found overall higher levels of IL-13 than IFN-γ mRNA in the liver of chickens while our studies did not show a significant increase in frequencies of IFN-γ or IL-13-producing liver cells from infected birds. In another study, which analyzed cytokine mRNA expression by ISH, spleens and livers of infected chickens showed no distinct elevated levels of IFN-γ or IL-13 mRNA in comparison to controls [12]. On the contrary, we found significant differences in IFN-γ-producing CD4^+^ and CD4^−^CD8β^−^ splenocytes comparing infected and control chickens. A possible explanation for this discrepancy might be that ISH analyses identify actively IFN-γ mRNA expressing cells, whereas the in vitro re-stimulation assay applied in this study re-activates putative memory and effector T cells, as indicated by the extremely low frequencies of IFN-γ-producing cells found in medium cultures.

The liver is besides the cecum one of the main affected organs in the course of a *H.* *meleagridis* infection [32]. It was suggested that in chickens, an up-regulation of proinflammatory cytokines in the cecal tonsils limits the migration of parasites from the cecum towards the liver and explains the lower prevalence of liver lesions compared to turkeys [9]. In our analyses on IFN-γ-producing lymphocytes isolated from this organ only low frequencies of *H. meleagridis*-specific cells were found and differences between infected and control birds did not reach significance. This finding coincides with the fact that only one of the chickens in the infected group showed lesions in this organ and all birds were negative by IHC. Although it can be assumed that the negative findings by IHC were a result of the investigation of a non-*H. meleagridis* infested part of the organ, for the analysis of cytokine production lymphocytes of the entire liver were isolated (only a small portion of approximately 0.5 × 0.5 × 0.5 cm was devoted to IHC slide preparation). Hence, it is conceivable that local liver-resident *H. meleagridis*-specific T cells were barely induced, whereas the spleen harbors re-circulating effector and memory T cells, which might contribute also to immune responses in the gut.

In summary, our study is the first that indicates that *H. meleagridis* infection in chickens induces a systemic T-cell related immune response against *H. meleagridis* that is dominated by IFN-γ-producing CD4^+^ and CD4^−^CD8β^−^ splenocytes, whereas no hints for an IL-13 mediated type-2 immune response were found. With the established functional assays, we aim to address in future studies a comparison of the cytokine production in chickens infected with virulent *H. meleagridis* cultures and chickens vaccinated with attenuated *H. meleagridis* cultures as well as vaccinated and challenged birds. This will include a more detailed T-cell phenotyping, addressing specifically CD4^+^, CD8^+^ and γδ T cells.

## Supplementary information

**Additional file 1. Design of the animal infection experiment.** Twelve 28-day-old chickens were equally distributed to an infected and a control group at the day of infection. The birds were infected via the oral and cloacal route with an equally split inoculum of 6 × 10^5^ virulent *H. meleagridis* cells (23 passages) in combination with 6 × 10^6^ CFU *E. coli*, strain DH5α (infected group, *n* = 6). Birds of the control group (*n* = 6) were sham-infected with the *E. coli* strain DH5α (1 × 10^8^ CFU) only. For organ collection, three birds from each group were sacrificed on 3 consecutive days 2 weeks pi (X symbol) and 5 weeks pi, respectively.

**Additional file 2. Transfection of HEK293T cells with chicken IL-13 for scrutiny of the IL-13 mRNA PrimeFlow RNA**^**TM**^ **Assay.** It contains a detailed methodological description on the transfection of HEK293T cells for the ectopic expression of chicken IL-13.

**Additional file 3. Suitability of chicken IFN-γ-specific monoclonal antibodies specific for intracellular cytokine staining.** A panel of six mAbs with either mouse IgG1 isotype (2B7, 11G5, 7E3, 12F12), or mouse IgG2a isotype (12F7) and mouse IgG2b isotype (12D4) was tested on PMA/ionomycin stimulated splenocytes. For each antibody, results are shown for the optimal quantity (clone 12F7: 150 ng, 2B7: 50 ng, 11G5: 12.5 ng, 7E3: 100 ng, 12D4: 250 ng, 12F12: 100 ng), initially identified in experiments with serial dilutions. Goat-anti-mouse isotype specific RPE-conjugated antibodies were applied afterwards for fluorescence labelling. Cells were pre-gated as described in Additional file 4A. Results are representative of four experiments with splenocytes from three different chickens.

**Additional file 4. Gating strategy for lymphocytes from spleen and liver in multicolor flow cytometry.** For lymphocytes subjected to intracellular IFN-γ staining (A) and PrimeFlow^TM^ RNA Assay (Thermo Fisher Scientific) staining for IL-13 mRNA (B) a time gate as well as FSC-H/FSC-W and SSC-H/SSC-W doublet discrimination gates were applied consecutively. Lymphocytes were then selected within a FSC-A/SSC-A plot followed by a dead cell exclusion gate using the Fixable Viability Dye eFluor^®^ 780. (A) Frequencies of IFN-γ^+^ cells within CD4^+^, CD8β^+^ and CD4^−^CD8β^−^ subgates were determined. (B) Percentages of IL-13 mRNA^+^ cells were determined within total live lymphocytes after excluding cells stained with putative dye aggregates in the CD4/CD8β plot. The gating strategy is shown for splenocytes from representative experiments and was applied for both organs from all birds.

**Additional file 5. IL-13 mRNA staining in HEK293T cells by PrimeFlow**^**TM**^ **RNA Assay (Thermo Fisher Scientific).** (A) Gating strategy for HEK293T cells in multicolor flow cytometry. After applying a time gate transfected cells were selected within a FSC-A/SSC-A plot followed by a dead cell exclusion gate using the Fixable Viability Dye eFluor^®^ 506. Frequencies of IL-13 mRNA^+^ cells within live HEK293T cells were determined. (B) HEK293T cells were transfected with the pFLAG-CMV2 expression vector including a chicken IL-13 DNA insert (upper row) or a porcine IgE insert (lower row). Cells were stained with the IL-13 target probe and label probe (right panel) or with the label probe only (left panel). Percentages of IL-13 mRNA^+^ cells are indicated above the gate. Results are representative of two separate transfection experiments.

**Additional file 6. Frequencies of cytokine-producing lymphocyte subsets for all investigated organs and stimulation variants.** Frequencies of cytokine-producing lymphocyte subsets for all investigated organs and stimulation variants are given in this table. In addition, all calculated *E. coli* corrected values for control and infected birds are listed.

**Additional file 7. Influence of different** ***H. meleagridis*** **concentrations on the frequency of IFN-γ-producing CD4**^**+**^ **splenocytes.** Intracellular cytokine staining for IFN-γ was performed following 18 h antigen specific re-stimulation either with *H. meleagridis* at 5 × 10^4^/mL and *E. coli* (9.4 × 10^6^ CFU/mL) or a 10-fold lower concentration of *H. meleagridis* (5 × 10^3^/mL) and *E. coli* (9.4 × 10^5^ CFU/mL). Plots on the left of each stimulation variant compare frequencies of IFN-γ-producing CD4^+^ cells after combined *H. meleagridis*/*E. coli* stimulation or stimulation only with *E. coli* in infected and control chickens. Plots on the right compare frequencies of IFN-γ-producing CD4^+^ cells between infected and control chickens after stimulation with *H. meleagridis*/*E. coli* antigen with or without correction for the response against *E. coli* alone. Each symbol represents one bird, black and red colored symbols show birds sacrificed 2 weeks pi and 5 weeks pi, respectively, as percent of total CD4^+^ splenocytes. Asterisks indicate different *p*-values: *p ≤ 0.05, and **p ≤ 0.01.

**Additional file 8. Frequencies of IFN-γ-producing CD4**^**+**^ **and CD4**^**−**^**CD8β**^**−**^ **cells in the liver.** The upper panel shows frequencies of IFN-γ-producing CD4^+^ and CD4^−^CD8β^−^ cells after PMA/ionomycin or *H. meleagridis*/*E. coli* stimulation compared to medium or *E. coli*-only stimulation in control and infected birds. The lower panel compares frequencies of IFN-γ-producing CD4^+^ and CD4^−^CD8β^−^ cells after stimulation with PMA/ionomycin, *H. meleagridis*/*E. coli* or after correction for *E. coli* between infected and control birds. Each symbol represents one bird, black and red colored symbols show birds sacrificed 2 weeks pi and 5 weeks pi, respectively, as percent of total CD4^+^ or CD4^−^CD8β^−^ intrahepatic lymphocytes. Asterisks indicate *p*-value: *p ≤ 0.05.

**Additional file 9. Summary of significant differences between cytokine-producing lymphocyte subsets isolated from control and** ***H. meleagridis*** **infected chickens.** In case of *H. meleagridis*/*E. coli* stimulated results only significance of *E. coli* corrected values are displayed. Asterisks indicate *p*-value levels (*p ≤ 0.05, and **p ≤ 0.01).
