# Supplementary figures and images for: Cytokine production and phenotype of Histomonas meleagridis-specific T cells in the chicken
Source: Vet Res. 2019 Dec 5;50:107. doi: 10.1186/s13567-019-0726-z (PMC6896354; doi:10.1186/s13567-019-0726-z)

## Slide 1
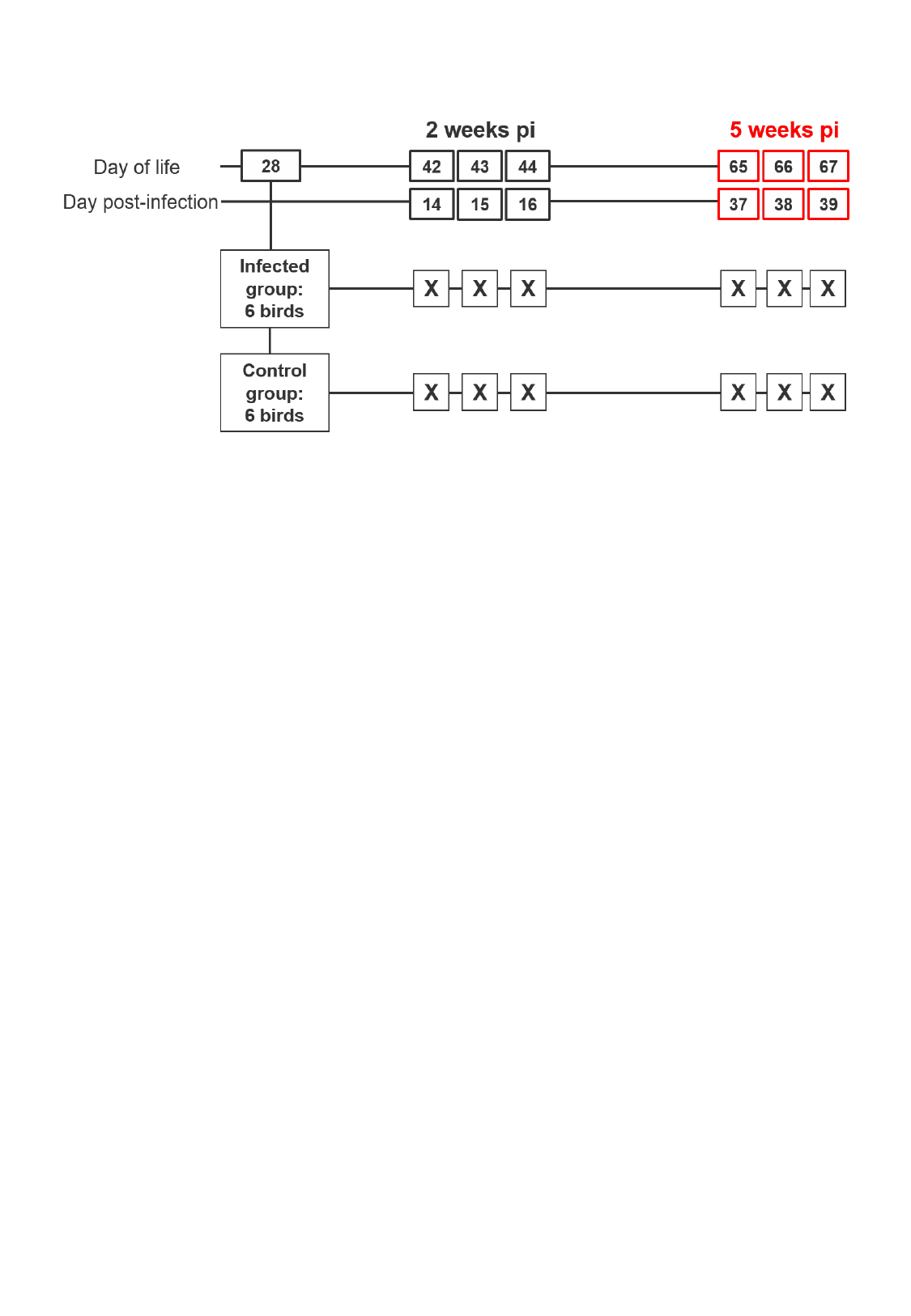

Supplement: Supplementary file 1 — Additional file 1. Design of the animal infection experiment. Twelve 28-day-old chickens were equally distributed to an infected and a control group at the day of infection. The birds were infected via the oral and cloacal route with an equally split inoculum of 6 × 10^5^ virulent H. meleagridis cells (23 passages) in combination with 6 × 10^6^ CFU E. coli, strain DH5α (infected group, n = 6). Birds of the control group (n = 6) were sham-infected with the E. coli strain DH5α (1 × 10^8^ CFU) only. For organ collection, three birds from each group were sacrificed on 3 consecutive days 2 weeks pi (X symbol) and 5 weeks pi, respectively. [file 13567_2019_726_MOESM1_ESM.pptx]

## Slide 1
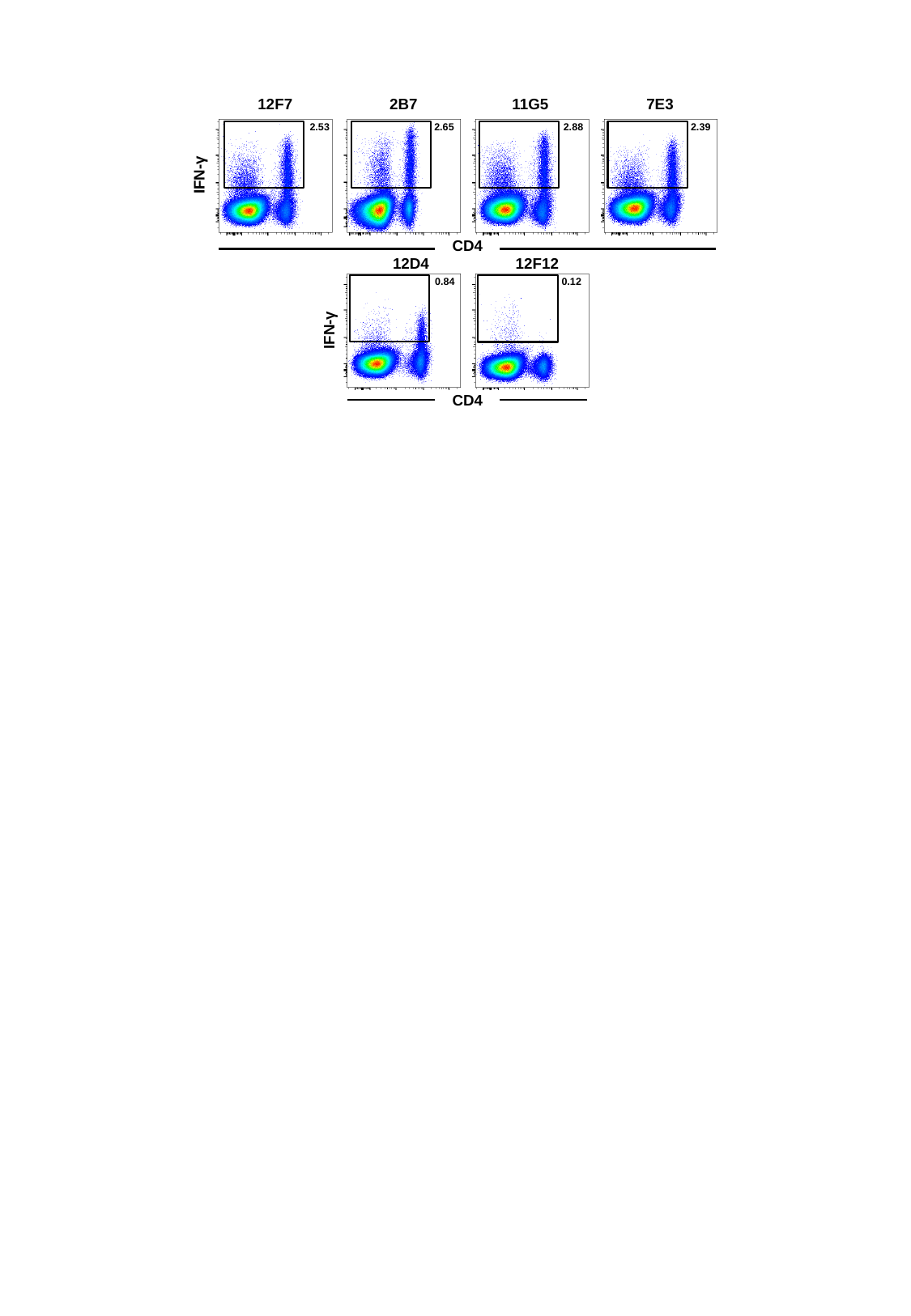

12F7
2B7
11G5
7E3
2.53
2.65
2.88
2.39
IFN-γ
CD4
12D4
12F12
0.84
0.12
IFN-γ
CD4

Supplement: Supplementary file 3 — Additional file 3. Suitability of chicken IFN-γ-specific monoclonal antibodies specific for intracellular cytokine staining. A panel of six mAbs with either mouse IgG1 isotype (2B7, 11G5, 7E3, 12F12), or mouse IgG2a isotype (12F7) and mouse IgG2b isotype (12D4) was tested on PMA/ionomycin stimulated splenocytes. For each antibody, results are shown for the optimal quantity (clone 12F7: 150 ng, 2B7: 50 ng, 11G5: 12.5 ng, 7E3: 100 ng, 12D4: 250 ng, 12F12: 100 ng), initially identified in experiments with serial dilutions. Goat-anti-mouse isotype specific RPE-conjugated antibodies were applied afterwards for fluorescence labelling. Cells were pre-gated as described in Additional file 4A. Results are representative of four experiments with splenocytes from three different chickens. [file 13567_2019_726_MOESM3_ESM.pptx]
