# Supplementary material for: Cytokine production and phenotype of Histomonas meleagridis-specific T cells in the chicken
Source: Vet Res. 2019 Dec 5;50:107. doi: 10.1186/s13567-019-0726-z (PMC6896354; doi:10.1186/s13567-019-0726-z)
Supplement: Supplementary file 2 — Additional file 2. Transfection of HEK293T cells with chicken IL-13 for scrutiny of the IL-13 mRNA PrimeFlow RNATM Assay. It contains a detailed methodological description on the transfection of HEK293T cells for the ectopic expression of chicken IL-13. [file 13567_2019_726_MOESM2_ESM.docx]

**Additional file 2: Transfection of HEK293T cells with chicken IL-13 for scrutiny of the IL-13 mRNA PrimeFlow RNA Assay**

For extraction of chicken IL-13 mRNA, PMA (50 ng/mL) and ionomycin (500 ng/mL; both Sigma-Aldrich) stimulated (2 h at 41 °C in 5% CO_2_) splenocytes isolated from a 72-week-old chicken were used. Lymphocyte isolation and stimulation was performed as in Materials and Methods. After harvest of stimulated cells, total RNA was extracted by using the Direct-zol RNA MiniPrep Kit (Zymo Research Corp., CA, USA) according to the manufacturer’s instructions, followed by RNA concentration measurement on the NanoDrop 2000c (Thermo Fisher Scientific). Subsequently, 500 ng total RNA were subjected to oligo-dT primed cDNA synthesis using the SuperScript^TM^ II Reverse Transcriptase (Thermo Fisher Scientific) according to the manufacturer’s instructions. For subcloning IL-13 into the pFLAG-CMV2 vector, appropriate restriction sites had to be introduced by proof-reading RT-PCR reactions using a EcoRI-tagged forward primer together with a XbaI-tagged reverse primer. Therefore, RT-PCR reactions were set-up in a total volume of 50 µL by combining 2 µL cDNA, 10 pM of each primer (Eurofins Genomics GmbH, Ebersberg, Germany), 1.25 U KAPAHiFi^TM^ Polymerase (Thermo Fisher Scientific), 10 µL 5x PCR buffer (KAPAHiFi^TM^ High Fidelity Buffer containing MgCl_2_ at a 1x concentration of 2.0 mM; Thermo Fisher Scientific), 1.5 µL dNTP mix (10 mM each dNTP; Thermo Fisher Scientific) and 5 µL 10x CoralLoad (Qiagen GmbH, Hilden, Germany). PCR cycling conditions were 2 min at 95 °C, followed by 20 s at 94 °C, 15 s at 60 °C, 30 s at 72 °C, for 35 cycles, followed by a final extension step for 3 min at 72 °C, using a T-gradient thermal cycler (Biometra, Göttingen, Germany). Obtained IL-13 PCR products were run on 1.5% standard agarose gels and further gel purified using the QIAquick Gel Cleanup Kit (Qiagen) according to the manufacturer’s instructions. Note: DNA concentrations of consecutive samples were measured on the NanoDrop 2000c (Thermo Fisher Scientific). Purified PCR products were ligated into the pJET1.2/blunt cloning vector (GeneJET^TM^ PCR cloning kit; Thermo Fisher Scientific) by combining in a total volume of 20 µL, 14.4 ng of purified PCR product (= vector to insert molar ratio of 1:3), 10 µL 2x Rapid Ligation buffer, 50 ng pJET1.2/blunt cloning vector and 5 U T4 DNA Ligase. Next, 4 µL ligated IL-13 products were transformed into 50 µL competent *E. coli* cells (JM109; Promega, Madison, WI, USA) and incubated on ice for 20 min. Thereafter, samples were incubated for 1 min at 42 °C and put back on ice for ≥2 min. After adding 500 µL SOC medium (2% tryptone, 0.5% yeast extract, 10 mM NaCl, 2.5 mM KCl, 10 mM MgCl_2_, 10 mM MgSO_4_ and 20 mM glucose; Sigma-Aldrich) they were incubated for 60 min at 37 °C with shaking at 700 rpm. Finally, 150 µL each were plated onto two LB/Amp^100^ plates (2% tryptone, 0.5% yeast extract, 10 mM NaCl, 15 g/L bacterial agar) being supplemented with Ampicillin (100 µg/mL) and incubated overnight at 37 °C.

Successful ligation and transformation of the tagged IL-13 inserts was confirmed by gene-specific PCR reactions on randomly selected *E. coli* colonies. Therefore, 6.25 µL 2x TopTaq^®^ HotStart DNA Polymerase Master Mix (Qiagen), 2.5 pM each of the tagged IL-13 primer and 1.25 µL 10x CoralLoad were added to 4.5 µL of *E. coli* colonies being resuspended in 100 µL PCR-grade water. Cycling conditions were 5 min at 95 °C, followed by 30 cycles of 30 s at 95 °C, 30 s at 60 °C, 30 s at 72 °C, followed by a final extension step of 5 min at 72 °C using a T-Gradient thermal cycler. Colony PCR reactions were screened on 1.5% standard agarose gels and positively identified cell suspensions were inoculated into 3 mL LB/Amp^100^ medium (2% tryptone, 0.5% yeast extract, 10 mM NaCl, 100 µg/mL Ampicillin) and incubated overnight at 37 °C with shaking at 250 rpm. Following overnight incubation, pJET1.2-IL-13 plasmid DNA was extracted according to the protocol of the Plasmid Mini-Prep classic kit (Zymo Research Corp.). Purified plasmids were analyzed for correct insert size by BglII digest by combining 200 ng plasmid DNA, 1 µL 10x BglII buffer (Thermo Fisher Scientific), 1 µL 10x CoralLoad (Qiagen) and 10 U of BglII (Thermo Fisher Scientific) in a total volume of 10 µL. After 1 hour incubation at 37 °C, samples were run on 1.5% standard agarose gels and positive clones were sent for bidirectional sequencing (Eurofins Genomics) for further confirmation.

For generating IL-13 expression constructs, the IL-13 insert was subcloned via EcoRI/ XbaI restrictions sites into the pFLAG-CMV2 vector. In separate reactions, the IL-13 insert was retrieved from positive pJET1.2-IL-13 plasmids and compatible pFLAG-CMV2 vector overhangs were generated by restriction digest with EcoRI and XbaI. Therefore, in a total volume of 20 µL, 500 ng plasmid DNA, 6 µL 10x Tango buffer (Thermo Fisher Scientific), 2 µL 10x CoralLoad, 10 U of EcoRI and 20 U of XbaI (both Thermo Fisher Scientific) were combined and incubated overnight at 37 °C. On the next day, samples were run on 1.5% standard agarose gels to further gel purify the EcoRI/ XbaI tagged IL-13 insert and the pFLAG-CMV2 vector by using the QIAquick Gel Cleanup Kit according to the manufacturer’s instructions. Next, IL-13 inserts were ligated into the pFLAG-CMV2 vector at a vector to insert molar ratio of 1:3. Therefore, in a total volume of 20 µL, 2 µL 10x T4 DNA Ligase buffer (Thermo Fisher Scientific), 50 ng pFLAG-CMV2 vector, 19 ng of purified IL-13 insert and 10 U T4 DNA Ligase (Thermo Fisher Scientific) were combined and incubated overnight at 4 °C. Transformation of ligated pFLAG-CMV2-IL-13 expression constructs, subsequent screening and sequence confirmation of positive clones were performed as described above. To obtain sufficient DNA of the IL-13-expression constructs for the subsequent transfection experiments, 200 mL of transformed *E. coli* cell culture were grown overnight (16-21 h until OD_600_ = 2-4) and subjected to Plasmid Midiprep (PureYield™ Plasmid Midiprep System; Promega) following the manufacturer’s instructions. Finally, correct orientation and intact reading-frame of pFLAG-IL-13-CMV expression constructs were performed by bidirectional sequencing being followed by generating bacterial glycerol stocks of the plasmids for long-term storage.

Prior to transfection, HEK293T cells were cultivated in T75 cell culture flasks with DMEM supplemented with 1 mM sodium pyruvate, 100 U/mL penicillin, 0.1 mg/mL streptomycin (all PAN-Biotech) and 10% heat-inactivated FCS (Sigma-Aldrich). 2.8 × 10^6^ cells were seeded and incubated at 37 °C in 5% CO_2_ for 48 hours. At a confluence of 80% cells were transfected with pFLAG-CMV2-IL-13 or as control with an irrelevant porcine IgE insert in a pFLAG-CMV2 expression vector using the PolyFect^®^ transfection reagent (Qiagen) according to the manufacturer’s instructions. In brief, 15 µg of DNA (pFLAG-CMV2-IL-13 or pFLAG-IgE) were incubated with 130 µL PolyFect^®^ reagent for 10 min at room temperature. Subsequently, 280 µL of the DNA complex mix was added to the cells and incubated for 24 h at 37 °C with 5% CO_2_. After adding 5 mL pre-warmed trypsin (PAN-Biotech) for 10 min at 37 °C with 5% CO_2_, cells were washed and subjected to intracellular IL-13 mRNA staining using the PrimeFlow^TM^ RNA assay according to manufacturer’s instructions (Thermo Fisher Scientific). Briefly, cells were stained with Fixable Viability Dye eFluor^®^ 506 (Thermo Fisher Scientific) for 20 min at 4 °C. After incubations for fixation and permeabilization the chicken IL-13 target probe was added to the cells for two hours at 41 °C. Two amplification steps were followed by an incubation with the fluorescence labelled probe. Cells were subsequently analyzed by flow cytometry.

**Primers used in this study**

Primer name Primer sequence (5’→ 3’) Primer position (5’→ 3’) Accession Number or web resource

EcoRI-IL-13 *GAA TTC* G**AT G**CA CCG CAC ACT GAA GGC nt 1 to 20 (forward primer) NM_001007085

IL-13-XbaI *TCT AGA* **TCA** GTT TGC AGC TGT GGC CGA nt 397 to 417 (reverse primer) NM_001007085

pJET1.2-fw CGA CTC ACT ATA GGG AGA GCG GC nt 310 to 332 (forward primer) CloneJET™ PCR Cloning Kit Manual^1^

pJET1.2-rev AAG AAC ATC GAT TTT CCA TGG CAG nt 428 to 405 (reverse primer) CloneJET™ PCR Cloning Kit Manual^1^

N-CMV-30 AAT GTC GTA ATA ACC CCG CCC CGT TGA CGC nt 825 to 854 (forward primer) E7398 (Adgene Vector Database)^2^

C-CMV-24 TAT TAG GAC AAG GCT GGT GGG CAC nt 1080 to 1103 (reverse primer) E7398 (Adgene Vector Database) ^2^

EcoRI and XbaI restriction sites are in italics.

ATG start and TGA stop codons are indicated by bold letters.

^1^ https://www.thermofisher.com/

^2^ https://www.addgene.org/vector-database/2770/
