# Supplementary material for: Cytokine production and phenotype of Histomonas meleagridis-specific T cells in the chicken
Source: Vet Res. 2019 Dec 5;50:107. doi: 10.1186/s13567-019-0726-z (PMC6896354; doi:10.1186/s13567-019-0726-z)
Supplement: Supplementary file 4 — Additional file 4. Gating strategy for lymphocytes from spleen and liver in multicolor flow cytometry. For lymphocytes subjected to intracellular IFN-γ staining (A) and PrimeFlowTM RNA Assay (Thermo Fisher Scientific) staining for IL-13 mRNA (B) a time gate as well as FSC-H/FSC-W and SSC-H/SSC-W doublet discrimination gates were applied consecutively. Lymphocytes were then selected within a FSC-A/SSC-A plot followed by a dead cell exclusion gate using the Fixable Viability Dye eFluor® 780. (A) Frequencies of IFN-γ+ cells within CD4+, CD8β+ and CD4−CD8β− subgates were determined. (B) Percentages of IL-13 mRNA+ cells were determined within total live lymphocytes after excluding cells stained with putative dye aggregates in the CD4/CD8β plot. The gating strategy is shown for splenocytes from representative experiments and was applied for both organs from all birds. [file 13567_2019_726_MOESM4_ESM.pptx]

## Slide 1
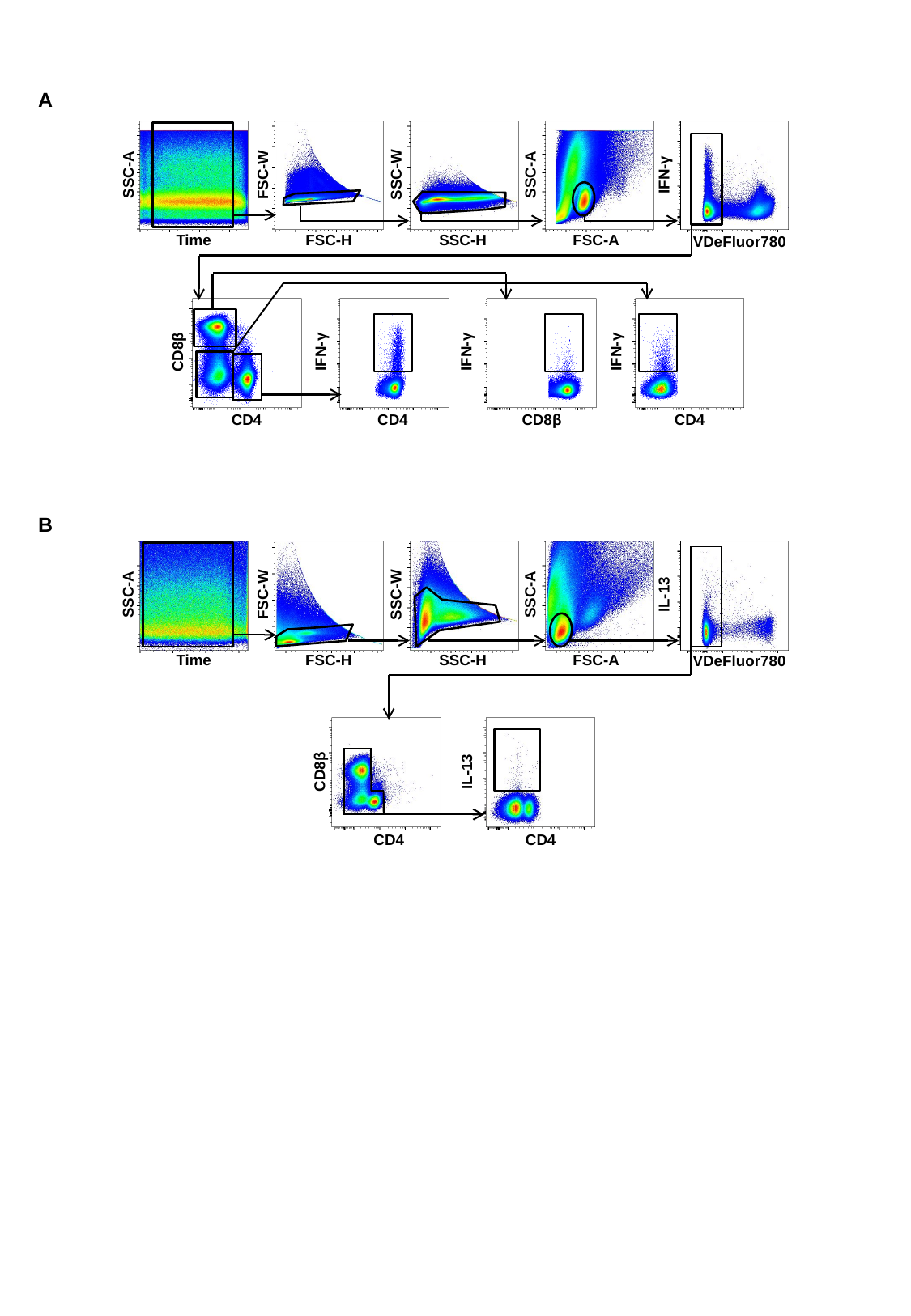

A
IFN-γ
SSC-W
SSC-A
SSC-A
FSC-W
SSC-H
Time
FSC-H
FSC-A
VDeFluor780
IFN-γ
IFN-γ
IFN-γ
CD8β
CD4
CD4
CD8β
CD4
B
IL-13
SSC-W
SSC-A
SSC-A
FSC-W
SSC-H
Time
FSC-H
FSC-A
VDeFluor780
IL-13
CD8β
CD4
CD4
