# Supplementary material for: Cytokine production and phenotype of Histomonas meleagridis-specific T cells in the chicken
Source: Vet Res. 2019 Dec 5;50:107. doi: 10.1186/s13567-019-0726-z (PMC6896354; doi:10.1186/s13567-019-0726-z)
Supplement: Supplementary file 5 — Additional file 5. IL-13 mRNA staining in HEK293T cells by PrimeFlowTM RNA Assay (Thermo Fisher Scientific). (A) Gating strategy for HEK293T cells in multicolor flow cytometry. After applying a time gate transfected cells were selected within a FSC-A/SSC-A plot followed by a dead cell exclusion gate using the Fixable Viability Dye eFluor® 506. Frequencies of IL-13 mRNA+ cells within live HEK293T cells were determined. (B) HEK293T cells were transfected with the pFLAG-CMV2 expression vector including a chicken IL-13 DNA insert (upper row) or a porcine IgE insert (lower row). Cells were stained with the IL-13 target probe and label probe (right panel) or with the label probe only (left panel). Percentages of IL-13 mRNA+ cells are indicated above the gate. Results are representative of two separate transfection experiments. [file 13567_2019_726_MOESM5_ESM.pptx]

## Slide 1
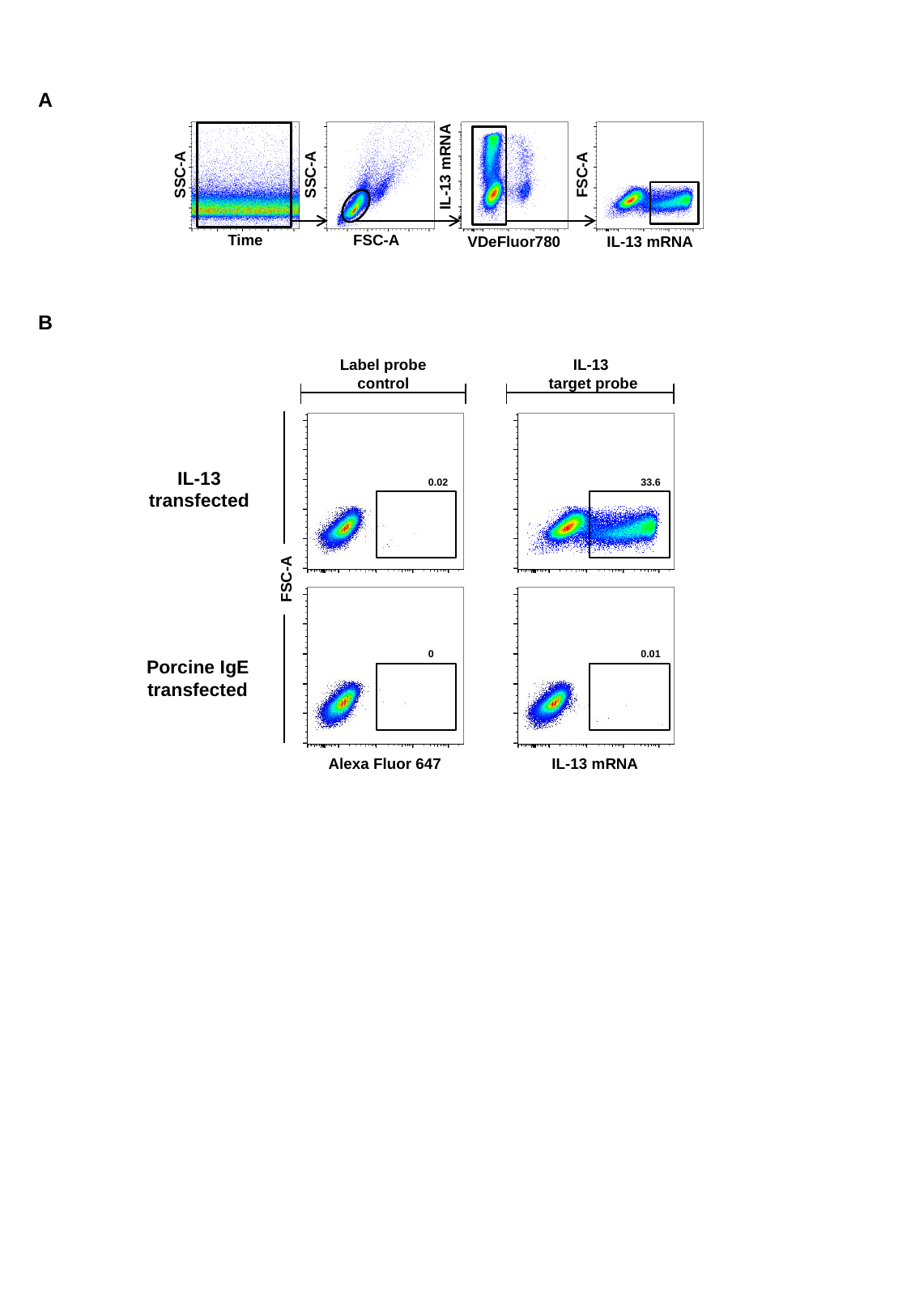

A
IL-13 mRNA
SSC-A
SSC-A
FSC-A
Time
FSC-A
VDeFluor780
IL-13 mRNA
B
Label probe control
IL-13
 target probe
IL-13 transfected
0.02
33.6
FSC-A
0
0.01
Porcine IgE transfected
Alexa Fluor 647
IL-13 mRNA
