# Supplementary material for: Cytokine production and phenotype of Histomonas meleagridis-specific T cells in the chicken
Source: Vet Res. 2019 Dec 5;50:107. doi: 10.1186/s13567-019-0726-z (PMC6896354; doi:10.1186/s13567-019-0726-z)
Supplement: Supplementary file 7 — Additional file 7. Influence of different H. meleagridis concentrations on the frequency of IFN-γ-producing CD4+ splenocytes. Intracellular cytokine staining for IFN-γ was performed following 18 h antigen specific re-stimulation either with H. meleagridis at 5 × 10^4^/mL and E. coli (9.4 × 10^6^ CFU/mL) or a 10-fold lower concentration of H. meleagridis (5 × 10^3^/mL) and E. coli (9.4 × 10^5^ CFU/mL). Plots on the left of each stimulation variant compare frequencies of IFN-γ-producing CD4+ cells after combined H. meleagridis/E. coli stimulation or stimulation only with E. coli in infected and control chickens. Plots on the right compare frequencies of IFN-γ-producing CD4+ cells between infected and control chickens after stimulation with H. meleagridis/E. coli antigen with or without correction for the response against E. coli alone. Each symbol represents one bird, black and red colored symbols show birds sacrificed 2 weeks pi and 5 weeks pi, respectively, as percent of total CD4+ splenocytes. Asterisks indicate different p-values: *p ≤ 0.05, and **p ≤ 0.01. [file 13567_2019_726_MOESM7_ESM.pptx]

## Slide 1
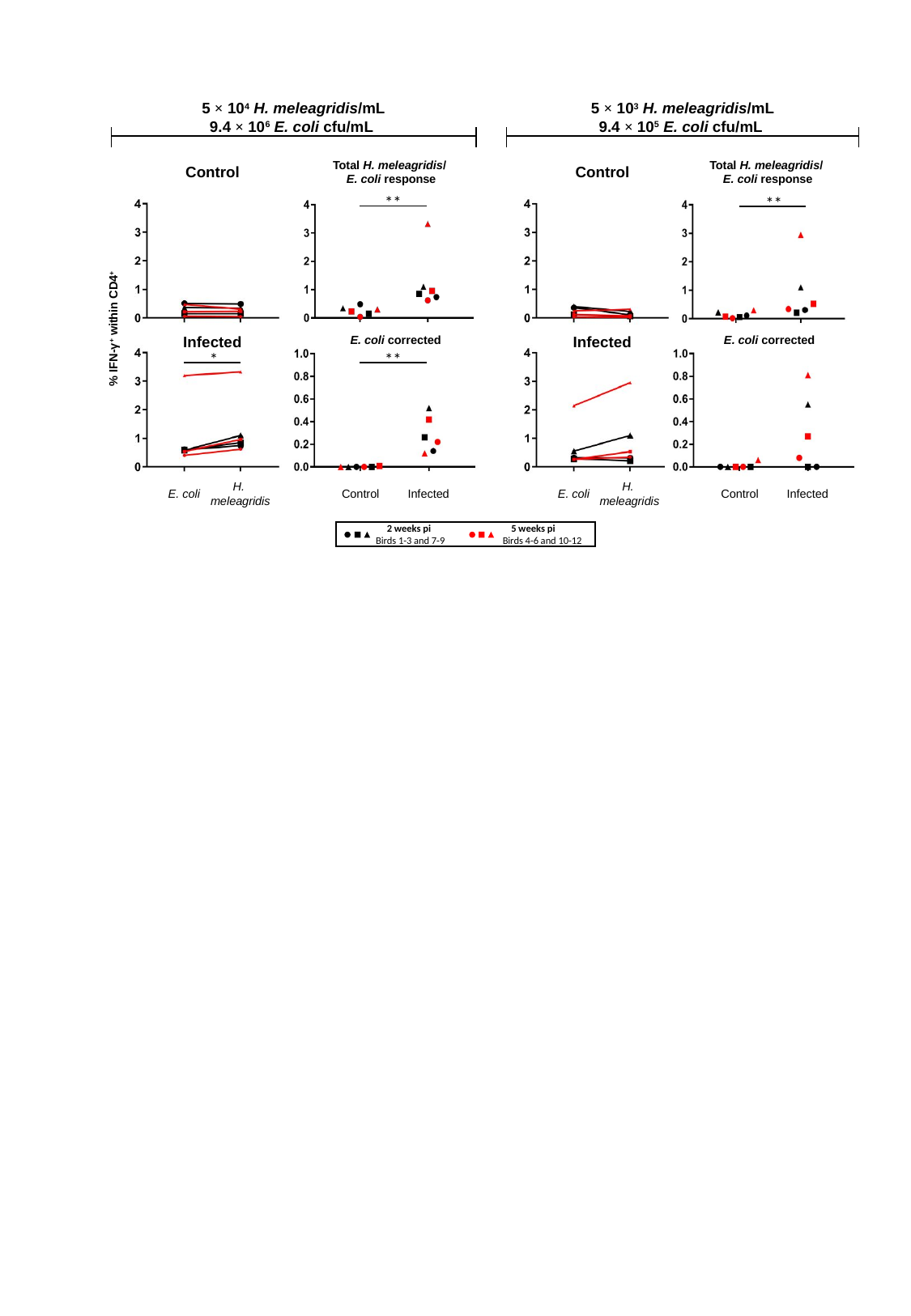

5 × 10^4^ H. meleagridis/mL
9.4 × 10^6^ E. coli cfu/mL
5 × 10^3^ H. meleagridis/mL
9.4 × 10^5^ E. coli cfu/mL
Total H. meleagridis/
E. coli response
Total H. meleagridis/
E. coli response
Control
Control
**
**
% IFN-γ+ within CD4+
Infected
E. coli corrected
Infected
E. coli corrected
*
**
H.
meleagridis
H.
meleagridis
E. coli
Control
Infected
E. coli
Control
Infected
 2 weeks pi 5 weeks pi
 Birds 1-3 and 7-9 Birds 4-6 and 10-12
