# Supplementary material for: Cytokine production and phenotype of Histomonas meleagridis-specific T cells in the chicken
Source: Vet Res. 2019 Dec 5;50:107. doi: 10.1186/s13567-019-0726-z (PMC6896354; doi:10.1186/s13567-019-0726-z)
Supplement: Supplementary file 8 — Additional file 8. Frequencies of IFN-γ-producing CD4+ and CD4−CD8β− cells in the liver. The upper panel shows frequencies of IFN-γ-producing CD4+ and CD4−CD8β− cells after PMA/ionomycin or H. meleagridis/E. coli stimulation compared to medium or E. coli-only stimulation in control and infected birds. The lower panel compares frequencies of IFN-γ-producing CD4+ and CD4−CD8β− cells after stimulation with PMA/ionomycin, H. meleagridis/E. coli or after correction for E. coli between infected and control birds. Each symbol represents one bird, black and red colored symbols show birds sacrificed 2 weeks pi and 5 weeks pi, respectively, as percent of total CD4+ or CD4−CD8β− intrahepatic lymphocytes. Asterisks indicate p-value: *p ≤ 0.05. [file 13567_2019_726_MOESM8_ESM.pptx]

## Slide 1
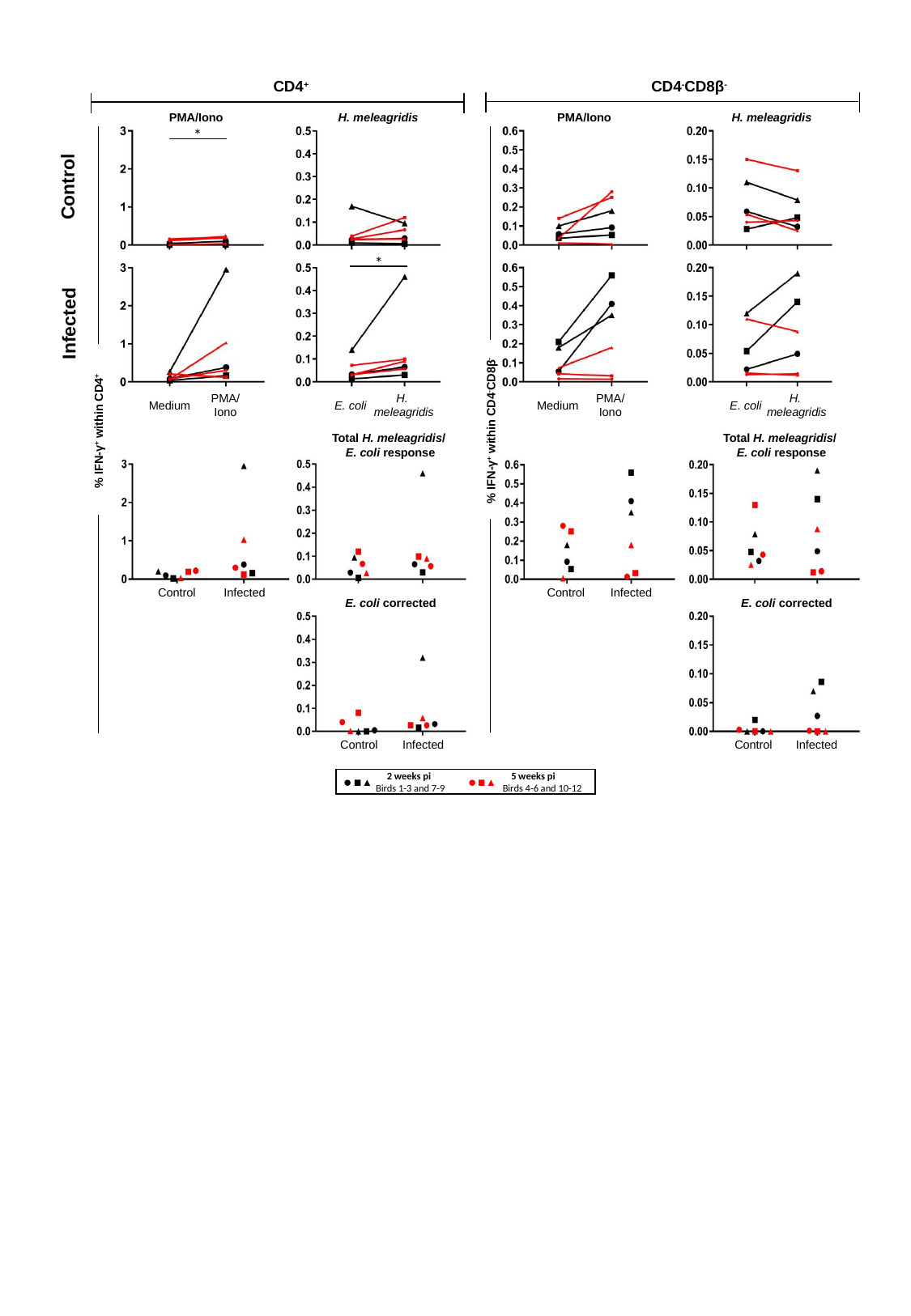

CD4+
CD4-CD8β-
PMA/Iono
H. meleagridis
PMA/Iono
H. meleagridis
*
Control
Infected
PMA/
Iono
H.
meleagridis
PMA/
Iono
H.
meleagridis
Medium
E. coli
Medium
E. coli
% IFN-γ+ within CD4-CD8β-
% IFN-γ+ within CD4+
Total H. meleagridis/
E. coli response
Total H. meleagridis/
E. coli response
Control
Infected
Control
Infected
E. coli corrected
E. coli corrected
Control
Infected
Control
Infected
 2 weeks pi 5 weeks pi
 Birds 1-3 and 7-9 Birds 4-6 and 10-12
*
