# Supplementary material for: Cytokine production and phenotype of Histomonas meleagridis-specific T cells in the chicken
Source: Vet Res. 2019 Dec 5;50:107. doi: 10.1186/s13567-019-0726-z (PMC6896354; doi:10.1186/s13567-019-0726-z)
Supplement: Supplementary file 9 — Additional file 9. Summary of significant differences between cytokine-producing lymphocyte subsets isolated from control and H. meleagridis infected chickens. In case of H. meleagridis/E. coli stimulated results only significance of E. coli corrected values are displayed. Asterisks indicate p-value levels (*p ≤ 0.05, and **p ≤ 0.01). [file 13567_2019_726_MOESM9_ESM.docx]

**Additional file 9. Summary of significant differences between cytokine-producing lymphocyte subsets isolated from *H. meleagridis* infected birds compared to control birds** (corrected values for *H. meleagridis* / *E. coli*).

|  | | Spleen | | | Liver | | |
| --- | --- | --- | --- | --- | --- | --- | --- |
|  |  | CD4^+^ | CD8β^+^ | CD4^-^CD8β^-^ | CD4^+^ | CD8β^+^ | CD4^-^CD8β^-^ |
| **IFN-γ** | PMA/Iono | ** | - | ** | - | - | - |
|  | *H. meleagridis* / *E. coli* | ** | - | - | - | - | - |
|  |  |  |  |  |  |  |  |
|  |  | Total lymphocytes | | |  | | |
| **IL-13** | PMA/Iono | - | | |  | | |
|  | *H. meleagridis* / *E. coli* | - | | |  | | |
|  |  |  |  |  |  |  |  |
